# Critical Assessment of Small Molecule Identification 2016: automated methods

**DOI:** 10.1186/s13321-017-0207-1

**Published:** 2017-03-27

**Authors:** Emma L. Schymanski, Christoph Ruttkies, Martin Krauss, Céline Brouard, Tobias Kind, Kai Dührkop, Felicity Allen, Arpana Vaniya, Dries Verdegem, Sebastian Böcker, Juho Rousu, Huibin Shen, Hiroshi Tsugawa, Tanvir Sajed, Oliver Fiehn, Bart Ghesquière, Steffen Neumann

**Affiliations:** 1 Eawag: Swiss Federal Institute for Aquatic Science and Technology, Überlandstrasse 133, 8600 Dübendorf, Switzerland; 2 Department of Stress and Developmental Biology, Leibniz Institute of Plant Biochemistry, Weinberg 3, 06120 Halle, Germany; 3 Department of Effect-Directed Analysis, UFZ: Helmholtz Centre for Environmental Research, Permoserstrasse 15, 04318 Leipzig, Germany; 4 Department of Computer Science, Aalto University, Konemiehentie 2, 02150 Espoo, Finland; 5 Helsinki Institute for Information Technology, Tekniikantie 14, 02150 Espoo, Finland; 6 West Coast Metabolomics Center and Genome Center, University of California Davis, 451 Health Sciences Drive, Davis, CA 95616 USA; 7 Chair of Bioinformatics, Friedrich-Schiller-University, Jena, Ernst-Abbe-Platz 2, 07743 Jena, Germany; 8 Department of Computing Science, University of Alberta, Edmonton, AB T6G 2E9 Canada; 9 Department of Chemistry, University of California Davis, One Shields Avenue, Davis, CA 95616 USA; 10 Metabolomics Expertise Center, Vesalius Research Center (VRC), VIB, KU Leuven – University of Leuven, 3000 Louvain, Belgium; 11 RIKEN Center for Sustainable Resource Science (CSRS), 1-7-22 Suehiro-cho, Tsurumi-ku, Yokohama, Kanagawa 230-0045 Japan; 12 Department of Biochemistry, Faculty of Sciences, King Abdulaziz University, Jeddah, Saudi Arabia

**Keywords:** Compound identification, *In silico* fragmentation, High resolution mass spectrometry, Metabolomics, Structure elucidation

## Abstract

**Background:**

The fourth round of the Critical Assessment of Small Molecule Identification (CASMI) Contest (www.casmi-contest.org) was held in 2016, with two new categories for automated methods. This article covers the 208 challenges in Categories 2 and 3, without and with metadata, from organization, participation, results and post-contest evaluation of CASMI 2016 through to perspectives for future contests and small molecule annotation/identification.

**Results:**

The Input Output Kernel Regression (CSI:IOKR) machine learning approach performed best in “Category 2: Best Automatic Structural Identification—*In Silico* Fragmentation Only”, won by Team Brouard with 41% challenge wins. The winner of “Category 3: Best Automatic Structural Identification—Full Information” was Team Kind (MS-FINDER), with 76% challenge wins. The best methods were able to achieve over 30% Top 1 ranks in Category 2, with all methods ranking the correct candidate in the Top 10 in around 50% of challenges. This success rate rose to 70% Top 1 ranks in Category 3, with candidates in the Top 10 in over 80% of the challenges. The machine learning and chemistry-based approaches are shown to perform in complementary ways.

**Conclusions:**

The improvement in (semi-)automated fragmentation methods for small molecule identification has been substantial. The achieved high rates of correct candidates in the Top 1 and Top 10, despite large candidate numbers, open up great possibilities for high-throughput annotation of untargeted analysis for “known unknowns”. As more high quality training data becomes available, the improvements in machine learning methods will likely continue, but the alternative approaches still provide valuable complementary information. Improved integration of experimental context will also improve identification success further for “real life” annotations. The true “unknown unknowns” remain to be evaluated in future CASMI contests.

**Electronic supplementary material:**

The online version of this article (doi:10.1186/s13321-017-0207-1) contains supplementary material, which is available to authorized users.

**Graphical abstract:**
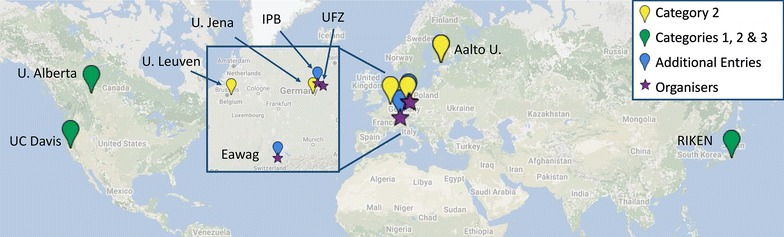
.

## Background

The Critical Assessment of Small Molecule Identification (CASMI) Contest [1] was founded in 2012 as an open contest for the experimental and computational mass spectrometry communities [2, 3]. Since then, CASMI contests have been held in 2013 [4], 2014 [5] and now in 2016, which is summarized in this article. The focus of CASMI has changed slightly with each contest, reflecting differences in focus of the organizers as well as the perceived interest and challenges in structure elucidation with mass spectrometry. CASMI is purely a research activity—there is no fee for participation but likewise also no prize money for the winners.

In 2016, Category 1 was “Best Structural Identification on Natural Products”, with 18 challenges available, a number achievable for both manual and automatic methods. Any methods could be used to submit entries and seven groups participated in this category. The outcomes of this category are presented separately [6] and reported here briefly for comparison purposes.

In contrast, Categories 2 and 3 were defined with 208 challenges in total. Candidate lists containing the correct solution were provided, along with training data for parameter optimization. These categories were specifically designed for automated methods, as no participant with a manual approach could be expected to invest so much time in solving all challenges. Category 2 was defined as “Best Automatic Structural Identification—*In Silico* Fragmentation Only”. The aim was to compare the different fragmentation approaches, ranging from combinatorial, to rule-based, to simulations; the use of mass spectral library searching or additional information was not allowed. In contrast, Category 3 was “Best Automatic Structural Identification—Full Information”. The same data files and candidate lists were provided as for Category 2, but any form of additional information could be used (retention time information, mass spectral libraries, patents, reference count, etc.). This was to assess the influence of additional information (hereafter termed metadata) on the results of the contest. Participants were required to detail their submissions in an abstract submitted with the results. The rules and submission formats were communicated on the CASMI rules website [7] prior to the release of the challenge data; the evaluation was automated provided the submission format passes all checks. In contrast to previous years, participants were allowed to submit up to three entries each, to evaluate the performance of different approaches. More details are given below.

This article summarizes Categories 2 and 3 of CASMI 2016, including organization, participation and additional post-contest analysis. Six external groups participated in these categories (see Graphical Abstract); 10 in total combined with the Category 1 participants, which is more than ever before.

## Methods

### Contest data for CASMI 2016

#### Mass spectra

All MS/MS spectra were obtained on a Q Exactive Plus Orbitrap (Thermo Scientific), with ${<}5$ ppm mass accuracy and nominal MS/MS resolving power of 35,000 at $m/z = 200$ using electrospray ionization (ESI) and stepped 20/35/50 nominal higher-energy collisional dissociation (HCD) energies. The spectra were obtained by measuring 22 mixes of authentic standards with the same liquid chromatography–mass spectrometry (LC–MS) method, in data-dependent acquisition mode using inclusion lists containing the $[\hbox {M}+\hbox {H}]^{+}$ (positive) and $[\hbox {M}-\hbox {H}]^{-}$ ion masses. Positive and negative mode data were acquired separately. Each mix contained between 10 and 94 compounds. A reversed phase column was used (Kinetex $\hbox {C}_{18}$ EVO, 2.6 μm, $2.1\times 50$ mm with a $2.1\times 5$ mm precolumn from Phenomenex). The gradient was (A/B): 95/5 at 0 min, 95/5 at 1 min, 0/100 at 13 min, 0/100 at 24 min (A = water, B = methanol, both with 0.1% formic acid) at a flow rate of 300 μL/min.

The MS/MS peak lists were extracted with RMassBank [8] using the ion mass and a retention time window of 0.4 min around the expected retention time and reported as absolute ion intensities. To obtain high-quality spectra, the data was cleaned and recalibrated to within 5 ppm using known subformula annotation [8], all other peaks without a valid subformula within 5 ppm of the recalibrated data were removed. All substances with double chromatographic peaks, different substances with identical spectra (detected via the SPectraL hASH (SPLASH) [9, 10]), MS/MS containing only one peak or with a maximum intensity below $1\times 10^{5}$ were excluded from the datasets. Substances that were measured multiple times (because they were present in more than one mix) in the same ionization mode were only included once, selected by higher intensity. MS/MS from positive and negative mode were included if the substance ionized in both modes. The final peak lists were saved in plain text format and Mascot Generic Format (MGF). All MS/MS spectra are now available on MassBank [11].

#### Candidates

The candidates were retrieved from ChemSpider via MetFrag2.3 [12] using the monoisotopic exact mass ±5 ppm of the correct candidate on February 14th, 2016. The SMILES from the MetFrag output were converted to standard InChIs and InChIKeys with OpenBabel (version 2.3.2) [13]. Candidates were removed if the SMILES to InChI conversion failed, all other candidates were retained without any additional filtering. The presence of the correct solution in the candidate list was verified and the lists were saved as CSV files.

#### Training and challenge datasets

The MS/MS spectra and corresponding candidates were split into training and challenge datasets, according to the spectral similarity to MassBank spectra (as many substances were already in MassBank). Challenge spectra were those where no MassBank spectrum was above 0.85 similarity (calculated with MetFusion [14]); all spectra where there was a match in MassBank above 0.85 were included in the CASMI training set. There were two exceptions: Alizarin, similarity 0.88 to laxapur (FIO00294), and anthrone, similarity 0.86 to phosphocreatine (KO003849), to ensure a sufficient number of natural products remained as challenges for Category 1 (see below). Many of the natural products in the mixes did not ionize well with the experimental setup used.

The challenge dataset consisted of 208 peak lists from 188 substances, 127 obtained in positive mode (all $[\hbox {M}{+}\hbox {H}]^{+}$) and 81 in negative mode (all $[\hbox {M}{-}\hbox {H}]^{-}$). The retention times for each substance was provided in a summary CSV file. The training dataset consisted of 312 MS/MS peak lists (from 285 substances), of which 254 were obtained in positive mode (all $[\hbox {M}+\hbox {H}]^{+}$) and 58 negative mode (all $[\hbox {M}{-}\hbox {H}]^{-}$). The identities and retention times of the substances in the training dataset were provided in a summary CSV file. All files were uploaded to the CASMI website [15]. Participants were asked to contact the organizers if they required additional formats.

To allow a comparison with manual approaches, Challenges 10–19 in Category 1 were a (re-named) subset of the dataset in Categories 2 and 3. The corresponding challenge numbers are given in Table 1.

**Table 1:** Overlapping challenges between Category 1 and Categories 2 and 3

Name	Category 1	Categories 2 and 3	Mode
Creatinine	Challenge-010	Challenge-084	Positive
Anthrone	Challenge-011	Challenge-162	Positive
Flavone	Challenge-012	Challenge-166	Positive
Medroxyprogesterone	Challenge-013	Challenge-184	Positive
Abietic acid	Challenge-014	Challenge-207	Positive
Estrone-3-($\upbeta$-d-glucuronide)	Challenge-015	Challenge-034	Negative
Alizarin	Challenge-016	Challenge-045	Negative
Thyroxine	Challenge-017	Challenge-048	Negative
Purpurin	Challenge-018	Challenge-054	Negative
Monensin	Challenge-019	Challenge-079	Negative

Information about the full scan (MS1) data was not originally provided for CASMI 2016, but was provided retrospectively for Challenges 10–19 in Category 1 upon request and post-contest for Categories 2 and 3 for another publication [16]. All data is now available on the CASMI website [15].

### Rules and evaluation

The goal of the CASMI contest was for participants to determine the correct molecular structure for each challenge spectrum amongst the corresponding candidate set, based on the data provided by the contest organizers. A set of rules were fixed in advance to clarify how the submissions were to be evaluated and ranked, to ensure that the evaluation criteria were transparent and objective. All participants were encouraged to follow the principles of reproducible research and accurately describe how their results were achieved in an abstract submitted with the results. Submission formats were defined in advance (described below) to satisfy the R scripts used to perform the automatic evaluation, results and web page generation. Test submissions could be submitted pre-deadline to check for issues; any post-deadline problems were resolved prior to the release of the solutions.

Participants could enter a maximum of three submissions per approach and category, provided they used these submissions to assess the influence of different strategies on the outcomes. The rationale and differences had to be detailed in the abstract. The *best overall performing submission* per participant was considered in declaring the winner(s). The submission requirements were an abstract file (per submission, see website for details) plus results files for each challenge to be considered in the contest. There was no explicit requirement to submit entries for all challenges. Valid challenge submissions were plain text, tab separated files with two columns containing the representation of the structure as the standard InChI or the SMILES code (column 1) and the score (column 2). To be evaluated properly, the score was to be non-negative with a higher score representing a better candidate.

For each challenge, the absolute rank of the correct solution (ordered by score) was determined. The average rank over all equal candidates was taken where two or more candidates had the same score. Due to inconsistencies with how participants dealt with multiple stereoisomers (and since stereoisomers amongst the candidates could not be separated with the analytical methods used), submissions were filtered post-submission to remove duplicate stereoisomers using the first block of the InChIKey. The *highest scoring* isomer was retained. The ranks were then compared across all eligible entries to declare the gold (winner), silver and bronze positions for each challenge. *Gold was awarded to the contestant(s) with the lowest rank among all contestants for that challenge*. This way, a winner could be declared even if no method ranked the correct candidate in the Top 1. Joint positions were possible in case of ties. The overall winner was determined using an Olympic medal tally scheme, i.e. the participants with the most gold medals per category won. The winners were declared on the basis of this automatic evaluation.

#### Additional scores

Further scores that were used to interpret the results included the mean and median ranks, Top X rank counts, relative ranking positions (RRPs, defined in [2]) and quantiles. The *Formula 1 Score*, based on the method used in Formula 1 racing [17] since 2010, is the sum of the Top 1 to 10 ranks of the correct candidates weighted by the scores 25, 18, 15, 12, 10, 8, 6, 4, 2 and 1. The *Medal Score* (as opposed to the per-challenge Gold Medal count used in CASMI to declare the winner) is the sum of weighted Top 1 ranks with 5 points (gold medal), Top 2 ranks with 3 points (silver) and Top 3 ranks (bronze) with 1. Non-integer ranks (due to equally-scoring candidates) were rounded up to the higher rank for calculating Top X, Formula 1 and medal scores (e.g. rank 1.5 was counted as 2).

### Participant methods

*Team Allen* (Felicity Allen, Tanvir Sajed, Russ Greiner and David Wishart) processed the provided candidates for Category 2 using CFM-ID [18]. CFM-ID uses a probabilistic generative model to produce an *in silico *predicted spectrum for each candidate compound. It then uses standard spectral similarity measures to rank those candidates according to how well their predicted spectrum matches the challenge spectrum. The original Competitive Fragmentation Model (CFM) positive and negative models were used, which were trained on data from the METLIN database [19]. Mass tolerances of 10 ppm were used, the Jaccard score was applied for spectral comparisons and the input spectrum was repeated for low, medium and high energies to form the CFM_orig entry. The CFM_retrain entry consisted of a CFM model trained on data from METLIN and the NIST MS/MS library [20] for the positive mode spectra. This new model also incorporated altered chemical features and a neural network within the transition function. Mass tolerances of 10 ppm were used, and the DotProduct score was applied for spectral comparisons. This model combined the spectra across energies before training, so only one energy exists in the output. The negative mode entries were the same as for CFM_orig.

CFM-ID was also used to submit entries for Category 3, by combining the above CFM-based score with a database score (DB_SCORE). For each hit in the databases HMDB [21], ChEBI [22], FooDB [23], DrugBank [24] and a local database of plant-derived compounds, 10 was added to DB_SCORE. The CFM_retrain+DB and CFM_orig+DB submissions were formed by adding the DB_SCORE for each candidate to the CFM_retrain and CFM_orig entries from Category 2, respectively.

*Team Brouard* (Céline Brouard, Huibin Shen, Kai Dührkop, Sebastian Böcker and Juho Rousu) participated in Category 2 using CSI:FingerID [25] with an Input Output Kernel Regression (IOKR) machine learning approach to predict the candidate scores [26]. Fragmentation trees were computed with SIRIUS version 3.1.4 [27] for all the molecular formulas present in the candidate set. Only the tree associated with the best score was considered. SIRIUS uses fragment intensities to distinguish noise and signal peaks, while the intensities were weighted lowly during learning (see [25, 26]). Different kernel functions were computed for measuring the similarities between either MS/MS spectra or fragmentation trees. Multiple kernel learning (MKL, see [28]) was used to combine the kernels as input for IOKR. In the CSI:IOKR_U submission, the same weight was associated with each kernel (uniform multiple kernel learning or “Uni-MKL”). In the CSI:IOKR_A submission the kernel weights were learned with the Alignf algorithm [29] so that the combined input kernel was maximally aligned to an ideal target kernel between molecules. In both submissions, IOKR was then used for learning a kernel function measuring the similarity between pairs of molecules. The values of this kernel on the training set were defined based on molecular fingerprints, using approximately 6000 molecular fingerprints from CDK [30, 31]. Separate models were trained for the MS/MS spectra in positive and negative mode. The method was trained using the CASMI training spectra, along with additional merged spectra from GNPS [32] and MassBank [33]. For the negative ion mode spectra, 102 spectra from GNPS and 714 spectra from MassBank were used. For the positive ion mode spectra, 3868 training spectra from GNPS were used. These training sets were prepared following a procedure similar to that described in [25].

The additional post-competition submission CSI:IOKR_AR used the same approach as CSI:IOKR_A, but the positive model was learned using a larger training set containing 7352 positive mode spectra from GNPS and MassBank. This training set was effectively the same as that used by Team Dührkop, with minor differences due to the pre-selection criteria of the spectra. The negative mode training set was not modified.

*Team Dührkop* (Kai Dührkop, Huibin Shen, Marvin Meusel, Juho Rousu and Sebastian Böcker) entered Category 2 with a command line version of CSI:FingerID version 1.0.1 [25], based on the original support vector machine (SVM) machine learning method. The peaklists were processed in MGF format and fragmentation trees were computed with SIRIUS version 3.1.4 [27] using the Q-TOF instrument settings. Trees were computed for all candidate formulas in the given structure candidate list; trees with a score ${<}80\%$ of the optimal tree score were discarded. The remaining trees were processed with CSI:FingerID. SIRIUS uses fragment intensities to distinguish noise and signal peaks, while the intensities are weighted lowly in CSI:FingerID (see [25]). Molecular fingerprints were predicted for each tree (with Platt probability estimates [34]) and compared against the fingerprints of all structure candidates (computed with CDK [30, 31]) with the same molecular formula. The resulting hits were merged together in one list and were sorted by score. A constant value of 10,000 was added to all scores to make them positive (as required in the CASMI rules). Ties of compounds with same score (and sometimes also with same 2D structure) were ordered randomly. The machine learning method was trained on 7352 spectra (4564 compounds) downloaded from GNPS [32] and MassBank [33]. All negative ion mode challenges were omitted due to a lack of training data; i.e. entries were only submitted for positive challenges. This formed the CSI:FID entry.

Team Dührkop submitted a second “leave out” entry, CSI:FID_leaveout, during the contest. Before the correct answer was known, the team observed that the top-scoring candidate matched a compound from the CSI:FID training set in 67 challenges, which could indicate that the method had memorized the training spectra. To assess the generalization of their method, the classifiers were retrained on the same training set, plus CASMI training spectra, but with these top scoring candidates removed. As this entry was “guesswork” and did not affect the contest outcomes, upon request Team Dührkop resubmitted a true “leave out” entry post-contest where all CASMI challenge compounds were removed from their training set (not just their “guess” based on top scoring candidates) prior to retraining and calculating the CSI:FID_leaveout results. For the sake of interpretation, only these updated “leave out” results are presented in this manuscript.

*Team Kind* (Tobias Kind, Hiroshi Tsugawa, Masanori Arita and Oliver Fiehn) submitted entries to Category 3 using a developer version (1.60) of the freely available MS-FINDER software [35, 36] combined with MS/MS searching and structure database lookup for confirmation (entry MS-FINDER+MD). MS-FINDER was originally developed to theoretically assign fragment substructures to MS/MS spectra using hydrogen rearrangement (HR) rules, and was subsequently developed into a structure elucidation program consisting of formula prediction, structure searching and structure ranking methods. For CASMI, an internal database was used to prioritize existing formulas from large chemical databases over less common formulas and the top 5 molecular formulas were regarded for structure queries. Each formula was then queried in the CASMI candidate lists as well as an internal MS-FINDER structure database. A tree-depth of 2 and relative abundance cutoff of 1% as well as up to 100 possible structures were reported with MS-FINDER. The final score was calculated by the integration of mass accuracy, isotopic ratio, product ion assignment, neutral loss assignment, bond dissociation energy, penalty of fragment linkage, penalty of hydrogen rearrangement rules, and existence of the compound in the internal MS-FINDER structure databases (see Additional file 1 for full details). MS-FINDER uses ion intensities in the relative abundance cutoff and isotopic ratio calculations, but not in candidate scoring.

Secondly, MS/MS search was used for further confirmation via the NIST MS Search GUI [37] together with major MS/MS databases such as NIST [20], MassBank of North America (MoNA) [38], ReSpect [39] and MassBank [33]. The precursor was set to 5 ppm and product ion search tolerance to 200 ppm. Around 100 out of the 208 candidates had no MS/MS information. For these searches, a simple similarity search without precursor information was also used, or the precursor window was extended to 100 ppm. Finally, those results that gave overall low hit scores were also cross-referenced with the STOFF-IDENT database of environmentally-relevant substances [40, 41] to obtain information on potential hit candidates. This step was taken because the training set consisted of mostly environmentally relevant compounds.

*Team Vaniya* (Arpana Vaniya, Stephanie N. Samra, Sajjan S. Mehta, Diego Pedrosa, Hiroshi Tsugawa and Oliver Fiehn) participated in Category 2 using MS-FINDER [35, 36] version 1.62 (entry MS-FINDER). MS-FINDER uses hydrogen rearrangement rules for structure elucidation using MS and MS/MS spectra of unknown compounds. The default settings were used; precursor *m/z*, ion mode, mass accuracy of instrument, and precursor type (given in CASMI) were used to populate the respective fields in MS-FINDER. Further parameter settings were: tree depth of 2, relative abundance cutoff of 1, and maximum report number of 100. Although relative abundance cutoffs were used to filter out noisy data, ion abundances were not used by MS-FINDER for calculation of either the score or rank of candidate structures. The default formula finder settings were used, except the mass tolerance, which was set to $\pm 5$ ppm mass accuracy as given by the CASMI organizers.

MS-FINDER typically retrieves candidates from an Existing Structure Database (ESD) file compiled from 13 databases, but this was disabled as candidates were provided. Instead, one ESD was created for each of the 208 challenges, containing the information from the candidate lists provided by the CASMI organizers. A batch search of the challenge MS/MS against the challenge candidate list (in the ESD) was performed on the top 500 candidates, to avoid long computational run times. Up to 500 top candidates structures were exported as a text file from MS-FINDER. Scores for automatically matching experimental to virtual spectra were ranked based on mass error, bond dissociation energy, penalties for linkage discrepancies, or violating hydrogen rearrangement rules. Final scores and multiple candidate SMILES were reported for 199 challenges for submission to CASMI 2016. Nine challenges could not be processed due to time constraints (Challenges 13, 61, 72, 78, 80, 106, 120, 133, 203). Full details on this entry, MS-FINDER and file modifications required are given in Additional files 1 and 2.

*Team Verdegem* (Dries Verdegem and Bart Ghesquière) participated in Category 2 with MAGMa+ [42], which is a wrapper script for the identification engine MAGMa [43]. For any given challenge, MAGMa+ runs MAGMa twice with two different parameter sets. A total of four optimized parameter sets exist (two for positive and two for negative ionization mode), which all differ from the original MAGMa parameters. Within one ionization mode, both corresponding parameter sets were each optimized for a unique latent molecular class. Following the outcome of both MAGMa runs, MAGMa+ determines the molecular class of the top ranked candidates returned by each run using a trained two-class random forest classifier. Depending on the most prevalent molecular class, one outcome (the one from the run with the parameters corresponding to the most prevalent class) is returned to the user. The candidate lists provided were used as a structure database without any prefiltering. MAGMa determines the score by adding an intensity-weighted term for each experimental peak. If a peak is explained by the *in silico *fragmentation process, the added term reflects the difficulty with which the corresponding fragment was generated. Otherwise, an “unexplained peak penalty” is added. Consequently, MAGMa returns smaller scores for better matches, and therefore the reciprocal of the scoring values was submitted to the contest. MAGMa was run with a relative *m* / *z* precision of 10 ppm and an absolute *m* / *z* precision of 0.002 Da. Default values were taken for all other options. MAGMa+ is available from [44].

To enable a comparison between MAGMa+ (entry MAGMa+) and MAGMa, entries based on MAGMa were submitted post-contest (entry MAGMa). MAGMa was run as is, without customization of its working parameters (bond break or missing substructure penalties). Identical mass window values as for MAGMa+ were applied (see above). Default values were used for all other settings. Again, the reciprocal of the scoring values was submitted to obtain higher scores for better matches.

#### Additional results

Additional results were calculated using MetFrag2.3 [12] to compare these results with the other methods outside the actual contest and to investigate the influence of metadata on the competition results. MetFrag command line version 2.3 (available from [45]) was used to process the challenges, using the MS/MS peak lists and the ChemSpider IDs (CSIDs) of the candidates provided. MetFrag assigns fragment structures generated *in silico *to experimental MS/MS spectra using a defined mass difference. The candidate score considers the mass and intensity of the explained peaks, as well as the energy required to break the bond(s) to generate the fragment. Higher masses and intensities will increase the score, while higher bond energies will decrease the score. The MetFrag submission consisted of the MetFrag fragmentation approach only. In the MetFrag+CFM entry the MetFrag and CFM-ID (version 2) [18] scores were combined. The CFM scores were calculated independently from Team Allen. Additionally, a Combined_MS/MS entry was prepared, combining six different fragmenters with equal weighting: CFM_orig, CSI:FID, CSI:IOKR_A, MAGMa+, MetFrag and MS-FINDER.

Several individual metadata scores were also prepared. A retention time prediction score was based on a correlation formed from the CASMI training set (submission Retention_time; +RT, see Additional file 1: Figure S1. The reference score (submission Refs) was the ChemSpiderReferenceCount, retrieved from ChemSpider [46] using the CSIDs given in the CASMI data. The MoNA submission ranked the candidates with the MetFusion-like [14] score built into MetFrag2.3, using the MoNA LC–MS/MS spectral library downloaded January 2016 [38]. The Lowest_CSID entry had candidates scored according to their identifier, where the lowest ChemSpider ID was considered the best entry.

The combined submissions to test the influence of different metadata on the results were as follows: MetFrag+RT+Refs, MetFrag+CFM+RT+Refs, MetFrag+CFM+RT+Refs +MoNA, Combined_MS/MS+RT+Refs and finally Combined_MS/MS+RT+Refs+MoNA. Full details of how all these submission were prepared are given in Additional file 1.

## Results

### CASMI 2016 overall results

The sections below are broken up into the official results of the two categories during the contest, shown in Table 2, followed by the post-contest evaluation and a comparison with all approaches from Category 1.

#### Category 2: *In silico* fragmentation only

The results from Category 2 are summarized in Table 2. The participant with the highest number of wins over all challenges (i.e. gold medals) was **Team Brouard** with 86 wins over 208 challenges (41%) for CSI:IOKR_A. **Team Dührkop** with CSI:FID (82 gold, 39%) and **Team Vaniya** with MS-FINDER (70 gold, 34%) were in second and third place, respectively. This clearly shows that the recent machine-learning developments have greatly improved the performance relative to the bond-breaking approaches and even CFM. The third place for MS-FINDER shows that it performs in quite a complementary way to the CSI methods. The performance of Team Dührkop is especially surprising considering that they did not submit any challenges in negative mode (due to a lack of training data).

**Table 2:**
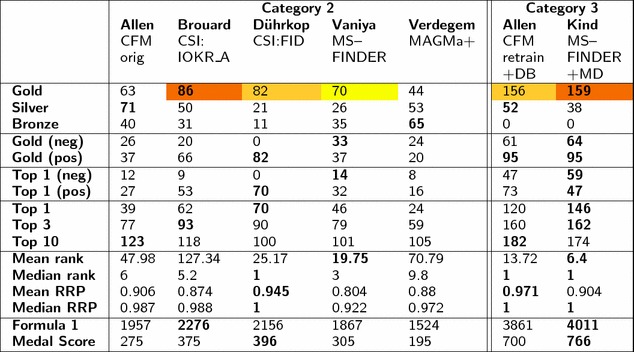
Results summary for Categories 2 and 3: medal tally and other statistics

The first, second and third place by “Gold medals” (used to declare CASMI winners) are highlighted in red, orange and yellow, respectively. The best value per statistic is marked in bold

Table 2 also includes the Top 1 (correct candidate ranked in first place), Top 3 (correct candidate amongst the top 3 scoring entries) and Top 10 entries per participant as well as the Formula 1 and Medal scores. The CSI:FID entry from Team Dührkop had the best Top 1 result (70, or 34%), followed by Team Brouard and Team Vaniya with 62 and 46 Top 1 candidates. This is an amazing improvement on previous contests and consistent with recent results [25], despite their use of larger candidate sets (PubChem instead of ChemSpider) and a slightly different ranking system. Very interesting to note is that all methods have the correct candidate in the Top 10 in ${\ge }49\%$ of cases, which is likewise a dramatic improvement for automatic annotation. CFM_orig had the most the correct candidates in the Top 10 (123 or 59%) and this is reflected in the Formula 1 Score, which weighted the CFM_orig performance ahead of MS-FINDER, despite their lower Top 1 ranks.

Separating the challenges into positive and negative modes revealed that Team Dührkop clearly led the positive mode predictions (82 wins/gold medals and 70 Top 1 candidates, versus 66 wins and 53 Top 1 candidates for Team Brouard). Both MS-FINDER (14 Top 1) and CFM_orig (12 Top 1) outperformed Team Brouard for negative mode (9 Top 1), showing that a greater amount of training data for negative spectra would likely improve the CSI methods in the future. The training set used by Team Brouard contained 7300 spectra for positive mode and only 816 negative mode spectra. The difference between positive and negative mode was less dramatic for the other approaches.

The results of Category 2 were dominated by the methods that use machine learning on large spectral databases (GNPS [32], MassBank [33], METLIN [19] and NIST [20]), namely Teams Brouard and Dührkop (CSI) and Allen (CFM). The great increase in data available for training these methods has led to the dramatic improvements in *in silico *methods seen in this contest—increasing the availability of open data will only improve this situation further! The performance of MS-FINDER, which does not use machine learning but instead chemical interpretation, is also particularly encouraging and below is shown to perform quite complementary to the machine learning methods. The influence of the training data was investigated during the contest by Teams Dührkop (CSI:FID_leaveout) and Allen (CFM_retrain); see Table 3. This was investigated for all approaches post-contest, discussed in “Machine learning approaches and training data” section.

#### Category 3: Full information

The results of Category 3, also summarized in Table 2, were extremely close considering the freedom given to the use of metadata in this Category. **Team Kind** was the winner with 159 gold (64 positive, 95 negative), closely followed by **Team Allen** on 156 gold (61 positive, 95 negative). Interestingly, the number of Top 1 ranks were very different, 146 (Team Kind) versus 120 (Team Allen); consistent with Category 2 CFM_orig had more Top 10 entries but fewer Top 1 and 3 entries than MS-FINDER. In this category the CFM_retrained model from Team Allen outperformed CFM_orig, which performed better in Category 2.

While very different approaches were used to obtain the “metadata”, the results of Category 3 clearly demonstrate the value of using metadata when identifying “known unknowns” as was the case in this contest where candidates were provided. This decision to provide candidates was taken deliberately to remove the influence of the candidate source on the CASMI results. The role of this “metadata” is discussed further below (Category 3: Additional Results). For true unknown identification the benefit of this style of metadata could be considerably reduced depending on the context, however this would have to be the subject of an alternative category in a future contest.

### Post-contest evaluation

While the best overall results per participant were used to declare the winners, each participant was able to submit up to three entries to the contest if they chose to assess the influence of different strategies on their outcome. This has revealed many interesting aspects that would otherwise have gone undetected with only one entry per participant, as in previous contests. To explore these further and take advantage of the automatic evaluation procedure offered in CASMI, several internal and post-contest entries were also evaluated, as described in the Methods section. The results of all these entries, including those run in the contest, are given in Table 3 for Category 2 and in Table 4 for Category 3.

#### Category 2: Additional results

The additional results for Category 2 (see Table 3) show that the retrained CSI:IOKR_AR entry from Team Brouard (using the more extensive CSI:FID training data plus negative mode results) would have outperformed their winning CSI:IOKR_A entry as well as the CSI:FID entry from Team Dührkop. The improvement with additional training data was dramatic for some challenges, e.g. Challenge 178 went from Rank 3101 with CSI:IOKR_A to rank 1 with CSI:IOKR_AR. Separating the Top 1 ranks into positive and negative mode (see Table 3) shows indeed that the performance for CSI:IOKR_AR and CSI:FID in positive mode was quite similar (69 vs. 70 wins, respectively), whereas all CSI methods are outperformed by MS-FINDER and CFM_orig in negative mode.

The MetFrag entry performed quite similarly to Team Verdegem (MAGMa+); as both are combinatorial fragmentation approaches this is not surprising. While the MetFrag+CFM entry improved these results dramatically, it was only slightly improved compared with the individual CFM entries of Team Allen. However, the improvement by combining the two fragmenters in negative mode was marked, increasing the Top 1 ranks from 9 (MetFrag) and 12 (CFM) to 20 (MetFrag+CFM). MS-FINDER still performed the best in negative mode of all the individual entries. MAGMa+ outperformed MAGMa in Top 1 and Top 3 entries.

**Table 3:** Results summary for additional Category 2 entries

	Allen		Brouard			Dührkop		Ruttkies		Vaniya	Verdegem	
	*CFM_orig*	CFM_retrain	*CSI:IOKR_A*	CSI:IOKR_AR*	CSI:IOKR_U	*CSI:FID*	CSI:FID_leaveout*	MetFrag*	MetFrag+CFM*	*MS−FINDER*	*MAGMa+*	MAGMa*
Top 1 Neg.	12	12	9	9	8	0	0	9	**20**	14	8	7
Top 1 Pos.	27	28	53	69	50	**70**	36	15	21	32	16	14
Top 1	39	40	62	**78**	58	70	36	24	41	46	24	21
Top 3	77	73	93	**102**	95	90	70	60	84	79	59	51
Top 10	123	116	118	**131**	118	100	88	108	127	101	105	106
Mean rank	47.98	44.53	127.3	95.09	123.3	25.17	52.02	51.92	33.97	**19.75**	70.79	70.24
Med. rank	6	7	5.25	4	5	**1**	3	8.75	6	3	9.8	9.8
Mean RRP	0.906	0.917	0.874	0.887	0.857	**0.945**	0.931	0.905	0.915	0.804	0.88	0.88
Med. RRP	0.987	0.985	0.988	0.993	0.98	**1**	0.995	0.98	0.991	0.922	0.972	0.969
Gold	53	52	73	**91**	70	74	41	32	51	61	35	31
Formula 1	1957	1900	2276	**2500**	2237	2156	1596	1593	2058	1867	1524	1463
Medal Sc.	275	269	375	**442**	371	396	252	198	292	305	195	175
Q_10	1	1	1	1	1	1	1	1	1	1	1	1.4
Q_25	2	2	1	1	1	1	1	3	2	1	3	3.5
Q_50	6	7	5.25	4	5	1	3	8.75	6	3	9.8	9.8
Q_75	36.25	27.63	55.5	36	78.75	6	17	37.88	25	17	66.1	64.5
Q_90	121.8	104.6	192.9	134.9	288.9	37.5	72.4	120.9	87.65	68.75	187.1	148.5

The column header of entries used in Table 2 are given in italics. The best value per statistic is marked in bold. * indicates internal and post-competition submissions. Med. = median. Q_X indicates Xth quantile

**Table 4:** Results summary for additional Category 3 entries

	Allen		Kind	Ruttkies		
	CFM orig +DB	*CFMretrain+DB*	*MS-FINDER+MD*	MetFrag+RT+Refs*	MetFrag+CFM+RT+Refs*	MetFrag+CFM+RT+Refs+MoNA*
Top 1	117	120	146	162	**163**	155
Top 3	159	160	162	**183**	180	182
Top 10	182	182	174	191	**199**	194
Mean rank	14	13.62	6.4	7.04	5.39	**4.25**
Median rank	**1**	**1**	**1**	**1**	**1**	**1**
Mean RRP	0.969	0.971	0.904	0.987	0.989	**0.990**
Median RRP	**1**	**1**	**1**	**1**	**1**	**1**
Gold	124	128	148	168	**174**	167
Formula 1	3798	3861	4011	4469	**4509**	4437
Medal score	687	700	766	855	**856**	840
Q_10	1	1	1	1	1	1
Q_25	1	1	1	1	1	1
Q_50	1	1	1	1	1	1
Q_75	3	3	2	1	1	2
Q_90	13.7	14.0	15.0	5.0	5.0	4.3

The column header of entries used in Table 2 are given in italics. The best value per statistic is marked in bold. * Indicates internal and post-competition submissions. Q_X indicates Xth quantile

#### Category 3: Additional results

The additional results for Category 3 (see Table 4) show that MetFrag+CFM+RT+Refs outperformed the other approaches both in terms of wins and the number of Top 1 ranks. Although adding MoNA to the mix resulted in a poorer performance, this was because spectral similarity was used to separate the training and challenge sets and the resulting MoNA weight was too optimistic for the challenges.

As these results are driven more by the metadata used than the fragmenter behind, a variety of entries were created to assess the contribution of the individual metadata aspects, as well as a “Combined Fragmenter” entry (Combined MS/MS) to remove the influence of the fragmentation method (see “Methods” for details). These results are given in Table 5. The Combined MS/MS entry outperformed all of the individual Category 2 entries, showing the complementarity of the different approaches. These also outperformed the MS library (MoNA) entry. The retention time prediction alone performed poorly, because this does not contain sufficient structural information to distinguish candidates, as demonstrated in Additional file 1: Figure S2. The lowest identifier strategy, which was used as a “gut feeling” decision criteria commonly in environmental studies before retrieval of reference information could be automated, takes advantage of the fact that well known substances were added to ChemSpider earlier and thus have lower identifiers. Surprisingly this still outperformed the combined fragmenters—but again this is highly dependent on the dataset. The references outperformed all individual metadata categories and even the combined fragmenters clearly. The influence of the metadata is discussed further in “Metadata and consensus identification” section.

**Table 5:** Contribution of Metadata to the results

	RT	MoNA	Lowest CSID	Refs	Combined MS/MS	Combined MS/MS+RT+Refs	Combined MS/MS+RT+Refs+MoNA
Top 1	1	70	113	143	82	**164**	**164**
Top 3	5	87	158	177	126	183	*187*
Top 10	20	104	177	**196**	166	194	195
Mean rank	504.5	238.3	37.7	**3.0**	13.4	3.9	3.7
Median rank	135	10.25	**1**	**1**	2	**1**	**1**
Mean RRP	0.576	0.780	0.959	**0.995**	0.955	0.990	0.991
Median RRP	0.630	0.977	**1**	*1*	0.998	**1**	**1**

The first four columns contain submissions formed using just one type of metadata, the “Combined MS/MS” column was formed by equally weighting all Category 2 entries from Table 2, while the last two columns combined this with retention time and references without and with MoNA, respectively

The best value per statistic is marked in bold

**Table 6:**
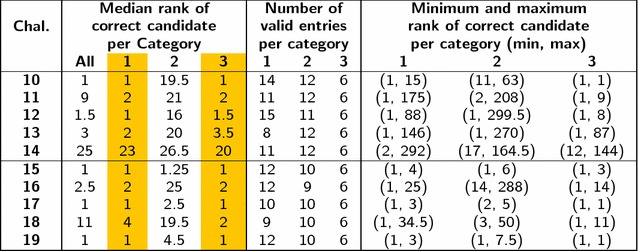
Comparison of Categories 1, 2 and 3 results for the overlapping challenges in Category 1

The median ranks of Categories 1 and 3 (highlighted) are remarkably similar

#### Comparison with results from Category 1

Challenges 10–19 in Category 1 were also present among the Category 2 and 3 challenges, as given in Table 1. The results for these challenges, separated by category, are summarized in Table 6 and visualized in Figures S3 and S4 in Additional file 1. Interestingly, this shows that the results of Categories 1 and 3 were remarkably comparable, while the ranks of Category 2, using only MS/MS data, were generally worse. Again, this shows that the incorporation of metadata in automated methods is essential to guide users to the identification for known substances—but misleading when assessing the performance of computational methods. As metadata cannot assist in the identification of true unknowns for which no data exists, more work is still needed to bring the performance of the *in silico *MS/MS identification methods (Category 2) closer to that of Categories 1 and 3. However, it is clear from this 2016 contest that much progress has been made with the new machine learning methods and—as observed above—continuing to improve the availability of training data will improve these further.

Interestingly, Challenge 14 (Abietic acid) was challenging for all participants in all categories; this was the only challenge in Category 1 where no participant had the correct answer in first place despite the fact that the challenge spectrum was very informative and the candidate numbers were relatively low (see Additional file 1: Figure S7).

## Discussion

### Visualization of CASMI results: clustering

**Fig. 1:**
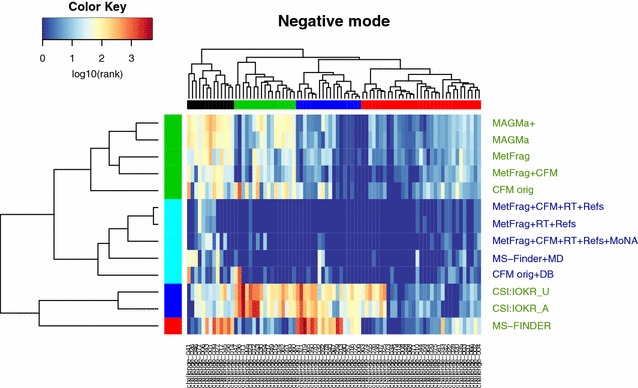
Heat Map of CASMI Challenges 1–81 (negative mode). Both Category 2 (*green labels* on the *right*) and 3 (*blue labels*) participants are included. Missing values (correct solution missed, or no submission for a challenge) were replaced with the number of candidates for that challenge. Ranks are log-scaled from good (*blue*) to poor (*red*). Team Dührkop was omitted as they did not submit for any challenge, while CSI:IOKR_AR and CFM_retrain were omitted as these were identical with their original submissions. An interactive version of this plot with legible challenge numbers is available from [47]

**Fig. 2:**
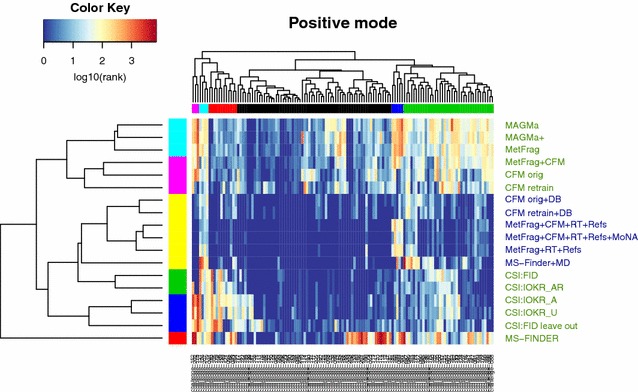
Heat Map of CASMI Challenges 82–208 (positive mode) both Category 2 (*green labels* on the *right*) and 3 (*blue labels*) participants are included. Missing values (correct solution missed, or no submission for a challenge) were replaced with the number of candidates for that challenge. Ranks are log-scaled from good (*blue*) to poor (*red*). Interactive version with legible challenge numbers available from [48]

To visualize the CASMI 2016 results together, a hierarchical clustering was performed. The heat map of the negative mode challenges (1–81, excluding Team Dührkop) can be seen in Fig. 1, while the heat map of the positive mode challenges (82–208) is given in Fig. 2. These are discussed below; in addition interactive plots are provided (see reference links provided in the captions) for readers to investigate these clusters in more detail. Corresponding clusters excluding challenges in the training sets are available in Additional file 1: Figures S5 and S6.

The dark blue areas in Fig. 1 indicate very good ranking results. It is clear for the negative spectra that the metadata (Category 3) really improved performance, with very few yellow or red entries for the Category 3 participants, which all grouped together in the cyan cluster (middle left), indicated by the dark blue participant names (middle right). What is also clear is that all methods were very good for most of the compounds in the red challenge cluster (shown at the top, right-most cluster). The combinatorial fragmenters and CFM also performed well on the dark blue challenge cluster (second cluster from right)—in contrast both MS-FINDER and the CSI:IOKR methods struggled for these challenges, shown with the yellow to red coloring in the heat map. MS-FINDER outperformed other Category 2 approaches in the green challenge cluster (second from left)—showing the complementarity of the different approaches. This is reinforced by the fact that MS-FINDER was split into a participant cluster on its own and also explains partially why the Combined MS/MS entry performed better than all individual participant entries. For the clusters of challenges (top), the mean candidate numbers per cluster were (left to right): black (611), green (1603), blue (1019) and red (380), compared with a mean overall of 816. Both the red (“good” overall performance) and black (“poor”) clusters have mean candidates below the overall mean, whereas the poorly performing green cluster had mean candidates well above the overall mean. Thus, candidate numbers are not the only driver of performance.

Looking at individual challenges, all machine learning approaches performed poorly for Challenge 36, which was a 3 peak spectrum of a substance typically measured in positive mode (see Additional file 1: Figure S8). The combinatorial approaches performed poorly for Challenge 41 (see Additional file 1: Figure S9), monobenzyl phthalate, where the main peak is a well-known rearrangement that is not covered by these approaches. For this challenge, both CSI:IOKR and MS-FINDER performed well, indicating that this substance is in the training data domain (many phthalate spectra are in the open domain) and that MS-FINDER interprets the spectrum beyond combinatorial methods. The compounds in the dark blue and green challenge clusters are likely not to be covered too well in the training data for CSI:IOKR. While it appears that MS-FINDER performs very poorly for some challenges, this is in fact an artifact of their submissions; for all the red entries in the heatmap, either the correct answer was absent from their submission (as they took only the top 500 candidates—this applied for 15 challenges) or no answer was submitted (5 challenges). In these cases the total number of candidates was used for the clustering. Removing the challenges where no submission was made from the clustering did not drastically alter any of the outcomes discussed above.

The positive mode cluster (Fig. 2) revealed an even darker blue picture (and thus generally very good results) than the negative mode cluster. The large dark blue patch in the middle of the heat map indicates that for the majority of challenges, largely those in the black challenge cluster (top, middle), both the metadata but also the more extensive training data in positive mode for the machine learning approaches ensured that many Top 1 ranks were achieved. This is also shown well in the green challenge cluster, where the improvements that the metadata and machine learning add beyond the combinatorial approaches can be seen moving down and getting darker from the generally yellow top right corner. As for negative mode, the mean candidate numbers per challenge cluster were calculated (left to right): magenta (5297), cyan (1029), red (886), black (1534), blue (978), green (722), with an overall mean of 1281. The performance for the magenta, cyan and blue clusters were all relatively “poor”, yet only the magenta cluster contained mean candidate numbers far above the overall mean. The combinatorial fragmenters performed poorly for the green cluster, which had mean candidate numbers below the overall mean. As mentioned above, candidate numbers are again not the only driver of performance. Investigations into other parameters that may influence the challenge clusters, such as number of peaks in the spectra, revealed similarly inconclusive results.

In contrast to negative mode, several participant clusters were formed in positive mode. The top two clusters contained the combinatorial fragmenters MAGMa, MAGMa+ and MetFrag, which clustered apart from the CFM-ID entries, either alone or in combination with MetFrag. Below this was one very large cluster with all Category 3 entries (metadata, yellow). This is followed by three smaller clusters, one in green with the two best CSI entries (CSI:FID and CSI:IOKR_AR), one blue cluster with the remaining CSI entries, followed by MS-FINDER by itself. Note that MS-FINDER still clustered by itself in both positive and negative mode, even when compensating for the challenges with no submission, as mentioned above. This is due in part to their strategy to only select the top 500—again for the vast majority of the red MS-FINDER entries in the heat map either the correct candidate was missing in the submission (29 challenges in positive mode), or no submission was made (4 challenges). However, their location in a separate cluster is also possibly due to the fact that MS-FINDER does indeed use a different approach to fragmentation than either the combinatorial fragmenters or the machine learning approaches.

The challenge clusters revealed some interesting patterns: four small clusters contained challenges that were problematic for different approaches. Most metadata-free methods performed poorly for the pink cluster (challenges 152, 202, 178); all approaches performed relatively poorly for the cyan cluster adjacent (challenges 131, 126, 207 and 119). The challenges in the red cluster were likely reasonably dissimilar to the other substances in the machine learning training sets, as the combinatorial fragmenters outperformed the CSI approaches clearly in this cluster. The machine learners performed well on the dark blue cluster (challenges 184, 168, 199, 92, 197), where surprisingly the metadata even failed the combinatorial fragmenters. Three of these (92, 168, 199) involve breaking an amide bond, which may be something for these approaches to investigate further. Challenge 197 is a fused N heterocycle with one fragment. Spectra of these challenges, with additional comments, are available in Additional file 1: Figures S7–S20.

### Visualization of CASMI results: candidate numbers and raw scores

Additional plots have been included in Additional file 1 to provide further visualization of the results. Additional file 1: Figure S21 shows the number of candidates for each challenge, ordered by the number of candidates versus the results for all CASMI entries (during and post-contest). Interestingly, fewer Top 1 entries and higher median/mean ranks were observed for the challenges with moderate candidate numbers (200–1000 candidates); lower median ranks and more Top 1 entries were observed for lower and higher candidate numbers. Additional file 1: Figures S22–S30 show the raw scores for selected submissions per participant and category, in order: MAGMa+, CSI:IOKR_A, CSI:FID, CFM_orig, CFM_retrain+DB, MS-FINDER, MS-FINDER+MD, MetFrag and MetFrag+CFM+RT+Refs+MoNA. These reveal interesting differences in the raw data behind each submission, including for instance the influence of training data availability on the positive and negative challenge results for CSI:IOKR_A, the metadata step function in CFM_retrain+DB as well as the effect of score scaling on MetFrag.

### Machine learning approaches and training data

The CASMI2016 results show very clearly how the training data influences the performance of different approaches. The difference in Top 1 positive mode ranks between CSI:IOKR_A, 62 and CSI:FID, 70 (see Table 2) were due to the different training sets used, the CSI:IOKR_AR results (retrained on the same data as CSI:FID) had 69 Top 1 ranks. The results for CSI:IOKR in negative mode were also generally worse than all other approaches, which shows that the decision of Team Dührkop not to submit entries due to a lack of training data was quite well justified (even though it likely cost them the overall contest “win” for Category 2).

Team Dührkop noted that there was a large overlap between the challenges and their training set and investigated this with the CSI:FID_leaveout entry (described in the methods). For the sake of interpretation in this manuscript, this entry was updated post-contest once the exact solutions were known to make it a true “leave out” analysis. Although the performance was reduced compared with CSI:FID (36 vs. 70 Top 1 ranks in positive mode), the CSI:FID_leaveout entry still had more Top 1 ranks than any other non-CSI method in the contest (for positive mode only).

Following the idea of Team Dührkop, the CASMI results were evaluated for all participants on only those challenges where no contestant had the correct candidate in their training sets. Teams Dührkop, Allen and Brouard provided comprehensive lists of their training sets. These were used to determine the overlap between all training sets and the CASMI challenges. The results over those challenges that were not in *any* training set (44 positive and 43 negative challenges) are given in Table 7.

**Table 7:** Global leaveout analysis for additional Category 2 entries—including only challenges where the correct answer was not in any training set

	Allen		Brouard			Dührkop		Ruttkies		Vaniya	Verdegem	
	*CFM_orig*	CFM_re-train	*CSI_IOKR_A*	CSI_IOKR_AR*	CSI_IOKR_U	*CSI:FID_orig*	CSI:FID_leaveout*	MetFrag*	MetFrag+CFM*	*MS−FINDER*	*MAGMa+*	MAGMa*
Top 1 Neg.	6	6	6	6	4	0	0	4	10	7	4	3
Top 1 Pos.	4	9	9	10	7	9	13	1	3	3	2	2
Top 1	10	15	15	**16**	11	9	13	5	13	10	6	5
Top 3	23	24	29	26	27	17	23	16	27	25	16	14
Top 10	46	40	45	46	40	25	32	39	47	38	35	35
Mean rank	52.57	64.05	106.5	97.84	99.92	52.81	41.48	68.38	37.16	28.7	76.75	100.4
Med. rank	10	12.5	8	10	12	7	3	14.5	8	7.5	23.5	20.5
Mean RRP	0.863	0.872	0.849	0.856	0.837	0.891	0.91	0.863	0.878	0.738	0.832	0.811
Med. RRP	0.966	0.961	0.963	0.967	0.956	0.981	0.993	0.942	0.972	0.806	0.924	0.902
Gold	18	21	23	**26**	19	11	17	7	18	18	10	9
F1 score	628	654	**735**	691	632	403	557	484	707	594	462	434
Medal Sc.	79	94	**105**	98	91	59	87	50	95	85	46	46

n = 43 (negative) and n = 44 (positive)

The best value for selected statistics is marked in bold

The general observations made on the full contest data are supported by this reduced dataset as well, despite the unsurprising fact that the results on this reduced dataset were generally worse than the official contest results (see Table 2). This demonstrates that, as expected, machine learning methods do better on compounds from within their training sets (for example, the percentage of maximum Top 1 ranks dropped from 34 to 18%). Although the median ranks were worse, the Top 10 ranks still remained around 40–50% for most methods. Cluster plots on this reduced dataset for negative and positive mode, given in the supporting information (Additional file 1: Figures S5, S6), show similar patterns to the cluster plots on the full dataset.

Interestingly, these results show that the CSI:FID_leaveout entry outperformed CSI:FID, while CSI:IOKR_A also outperformed CSI:IOKR_AR, the retrained dataset, also for some different scores—similar observations could be made for CFM_orig versus CFM_retrain. While this could be a potential sign for overfitting, this is a small dataset and some or all of these observations could be due to fluctuations in the data. Overfitting is a potential problem that developers, especially of non-standard machine learning methods should test for, *e.g.* by checking if their performance decreases significantly for compounds which are structural dissimilar to compounds in the training data. These results highlight just one means by which the choice of training set can influence the performance of automated methods. The training set can also impact challenge results in a range of other ways that are harder to disambiguate. One training set may be more or less compatible with the challenge set, even after common compounds are removed. This suggests the importance of assessing automated methods using the same training set, where at all possible.

### Metadata and consensus identification

**Fig. 3:**
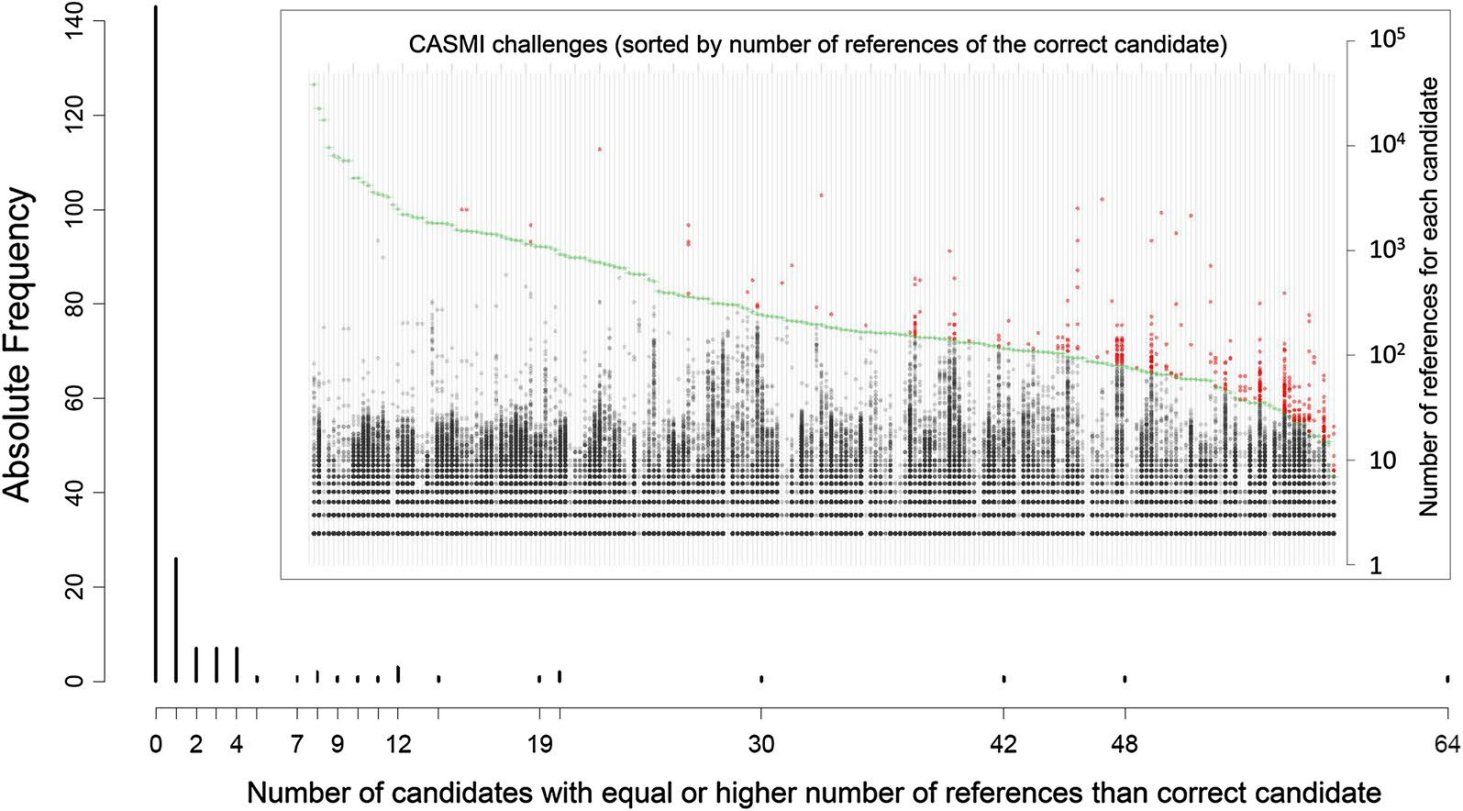
The distribution of references for CASMI 2016 candidates

**Fig. 4:**
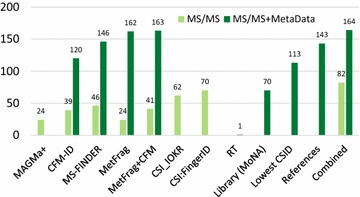
The influence of Metadata on CASMI 2016 first seven groups—*light green* MS/MS information only, i.e. Category 2. *Dark green* with metadata, i.e. Category 3 participants. Note these are plotted according to the number 1 ranks, not wins. Next 4 groups: *dark green* metadata only; Last group: *light green* is the equally-weighted combination of the six individual Category 2 entries and *dark green* is this plus metadata as shown in Table 5

The dataset for CASMI 2016 was predominantly well-known anthropogenic substances and as a result there are many distinct and highly referenced substances in the candidate lists. This is shown in the huge improvement that the metadata made to the ranking performance (Tables 4, 5). Figure 3 shows clearly that the vast majority of substances were either ranked first or second based purely on the reference count, with most other candidates having much lower counts. Figure 4 gives an overview of the contribution the metadata made to each approach based on the CASMI 2016 entries, merging team results in the case of MS-FINDER. In the environmental context, it is quite common to search an exact mass or formula in databases such as ChemSpider, where e.g. the highest reference count as well as the substance with the “lowest CSID” are often picked as the most promising hit in many cases, discussed e.g. in [49]. The success with these strategies would have been quite considerable with this dataset. However, for new (emerging) anthropogenic substances and transformation products of known chemicals, these strategies would not work so well as they would have neither a high reference count nor a low database identifier. This situation is also likely to be drastically different for natural products and metabolites, where many more closely-related substances or even isomers could be expected.

The metadata results in Category 3 show that the importance of the sample context cannot be ignored during identification, especially for studies looking to find well-known substances. This is also highlighted by the comparison with the approaches used in Category 1, where also manual and semi-automatic approaches were considered. The current reality is that most automated approaches still depend on retrieving candidates from compound databases containing known structures—i.e. the situation replicated in this CASMI contest. Compound databases such as the Metabolic *In Silico* Network Expansion Databases (MINEs) [50] could be used as alternative sources of candidates for predicted metabolites in the metabolomics context, but would have had limited relevance in this contest.

While metadata, the way it was used here, will not help in the case of true unknowns, there are two cases to consider for automated approaches at this stage. For “unknowns” that happen to be in a database almost accidentally (e.g. a to-date unknown transformation product), the automated fragmentation approaches are very useful, because these structures can be retrieved from substance databases. However for true “unknown unknowns” that are not in any database, fragmenters could only be used in combination with structure generation, which is still impractical with the quality of data and methods at this stage unless candidate numbers can be restrained sufficiently. These cases are often extremely difficult to elucidate using $\hbox {MS}^n$ alone and the information from additional analysis such as NMR will usually be necessary.

Stereoisomerism is another aspect of identification that was not covered in this contest. None of the current approaches are able to distinguish stereoisomers (even cis/trans isomers) using only MS/MS information for known unknowns. The evaluation of this contest addressed this by taking the best scoring stereoisomer and eliminating others (see “Methods”) to reduce the influence of stereoisomers on the ranking results. However, for electron ionization (EI) MS it is already possible to distinguish stereoisomers in some cases using ion abundances. This is an aspect that should be developed in the future for MS/MS once the spectrum generation is sufficiently reproducible to allow this. Coupling with suitable chromatography will potentially enhance the ability to distinguish between stereoisomers further.

### Evaluating methods and winner declaration

Contests such as CASMI always generate much discussion about how the winner was evaluated and declared; this years contest was no exception. A “contest” setting is different to the way individual methods compare their performance with others and this is the role of CASMI—to look at the approaches in different ways, relative to one another. One change in CASMI 2016 was to use the “average rank” instead of the “worst-case” rank to account for equal candidate scores, as participants pointed out that for previous contests one could add small random values to break tied scores and improve results in the contest. There will be several cases where candidates are indistinguishable according to the MS and it is important to capture this aspect in CASMI. While equal scores may make most chemical sense in these cases, computational methods deal with this differently; some report equal scores, others generate slightly different scores for effectively equal candidates. The average rank deals with this better than the “worst-case” rank, but can now disadvantage methods that report equal scores compared with others, as the chances are that at least one other method will beat it each time.

The criteria for declaring the winner in this contest was that the best performing participant(s), i.e. the winner, was defined per challenge and then the wins were added to determine the overall winner. This allows the declaration of a winner per challenge, irrespective of the actual performance (i.e. the winner could have rank 100, if all other participants were worse). The drawback of this approach is that it creates cross-dependencies between participants, i.e. the removal (or addition) of one participant completely changed the rank of the other participants. CFM likely suffered from this, as a machine-learning approach with similar training set coverage to CSI, which allowed the complementary approach of MS-FINDER to claim third place ahead of CFM. An alternative approach could be to look at this in terms of overall success and say that if a team had the correct structure as the 20th hit and other teams were even worse, none of the approaches were really sufficient to the task and nobody should then earn a ‘win’. This may reflect real structure elucidation cases better, where investigators would likely also consider the Top 3, Top 5, or maybe even Top 10 structures, but is perhaps not so good to declare a winner in a contest as some (difficult) challenges would have no “winner” and the performance of methods on difficult challenges is also an important aspect of the contest. This idea was investigated in this publication by also providing the Top 1, Top 3, Top 10 ranks per participant, as well as the Formula 1 Score (scaled Top 1–10 results) and Medal Score, where the medal count is based on Top 1, 2 and 3 ranks. The results of these metrics confirm the overall pattern observed in the contest: the two CSI teams outperformed all others in Category 2, followed by either MS-FINDER or CFM depending on exactly which score was used. In other words, the approaches have made fantastic progress, are complementary to one another but actually quite difficult to tell apart. Although 208 challenges is an order of magnitude in terms of challenge numbers above previous CASMIs, these numbers are still quite small and almost random differences between the methods resulted sometimes in large changes in the various scores, as shown with the different CSI entries.

### Participant perspectives

*Team Allen* submitted two alternative versions of CFM, the main difference being that for CFM_retrain version, additional training data was added from the 2014 NIST MS/MS database. While the addition of extra training data may have been expected to improve the results, this appears not to have been the case for this competition. One possible reason for this is that the additional data were generally of poorer (often integer) mass accuracy as compared to that used to train the original CFM model. This required a wider mass tolerance (0.5 Da) to be used during the retraining (compared to 0.01 Da previously), which may have hindered the training algorithm from accurately assigning explanations to peaks, and so modeling their likelihoods. This highlights that while the production of larger, more comprehensive data sets is likely crucial for better training of automated methods, the quality of these data sets is also very important. Most automated methods would likely benefit from training on cleaner data with better mass accuracies.

*Team Dührkop* investigated how CSI:FingerId compared with a direct spectral library search. A spectral library containing all structures and spectra used to train CSI:FingerId was created and searched with a 10 ppm precursor mass deviation. The resulting spectra were sorted via cosine similarity (normalized dot product), again with 10 ppm mass accuracy. Candidates were returned for 91 of the 127 (positive mode) challenges; the correct answer was contained in the library for 69 of these. The spectral library search correctly identified 63 of the 69 structures in total, 40 of these were “trivial” (the correct answer was the only candidate). On average, candidate lists for the spectral library search contained only 2.4 candidates, which was almost three orders of magnitude below the average CASMI candidate list of 1114 candidates. The cosine product between the challenge spectrum and the corresponding training spectrum of the same compound was only 0.76 on average; for one challenge it was below 0.01. For example, the cosine similarity between the spectrum for Challenge 202 (Pendimethalin) and the training spectrum was only 0.137, but it was still “correctly identified” as it was the only candidate with this precursor mass. This compound was correctly identified in the original CSI:FID submission, and ranked 569 for the CSI:FID_leaveout submission. This indicates that CSI:FingerId and other machine-learning approaches are capable of learning inherent properties from the mass spectra, beyond simple spectral similarity.

*Team Vaniya* The CASMI Category 2 contest was a reshuffling contest: potential structures were given to all participants, listing one to over 8000 potential structures for each challenge. These structures were within 5 ppm mass accuracy and often included different elemental formulas. Therefore, Category 2 was a ‘structure dereplication’ contest, finding the best structure within a pre-defined list of structures, not a completely open *in silico *test on all exhaustive structures in the chemosphere. In practical terms, it is important to note that an *in silico *software does not eliminate the time consuming aspects of data preparation, formatting, and interpretation. Counting the computing power and manual effort between two people, it took about 24 h to complete the 208 challenges for the MS-FINDER submission.

From Table 2, one could say that MS-FINDER was best based on the mean rank (19.75), but ranks lower than 10 are less relevant in reality. While MS-FINDER had almost 50% of the challenges within the top 10 ranks, so did every other software (or team). In reality, no chemist would use a software without any database or mass spectral library behind it. The importance of using *a priori* knowledge is seen by Team Allen’s submission that improved the Top 1 correct structure hits from 39 to 120 challenges in Category 3, a bit more than 50% of the challenges. Hence, we conclude that the glass is half full: if only *in silico *methods are used, some 50% of the challenges are within the top 10 hits within the structures given by the CASMI organizers. However, many challenges would score much higher if other metadata are used, e.g. constraining the search database to particular classes of compounds that can be expected for a specific study. Which parameters need to be optimized, and which *a priori* metadata should be used? Those questions may be answered in a more tailored future CASMI contest.

*Team Verdegem* participated in Category 2 of the CASMI 2016 contest with MAGMa+, which is a fast, plug-and-play method relying on combinatorial fragmentation without requiring a preliminary training phase for improved performance. The entire submission, including scripting for automation and single core calculations, took less than 1 day. MAGMa+ outperformed MAGMa, showing the use of the parameter optimization performed to improve several second and third ranked candidates to first place. MAGMa+ shared the best ranking for 44 of 208 challenges (see Table 2) and performed considerably better than other contestants for nine of those challenges (21, 32, 36, 40, 52, 61, 121, 157 and 189), indicating the relevance of the underlying algorithm.

Since MAGMa+ outperformed MAGMa according to some (e.g. number of gold medals, Top 1 and 3 ranks) but not all metrics, further more advanced parameter optimizations are planned to achieve a more global performance improvement. However, further improvements to the performance of MAGMa/MAGMa+ will require interventions of a different kind. The performance of MAGMa+ decreases with increasing candidate numbers (in this contest 1116 on average after the removal of duplicate stereoisomers), however, in case of smaller numbers, it starts to outperform some of the other methods [25, 42]. For untargeted metabolite identification in biological/biomedical setups, it is arguably more suitable to restrict the candidate structure database to those metabolites known to exist in the organism under study, e.g. using only the ${\approx }42{,}000$ metabolites currently present in the HMDB [21] for samples of human origin. This was noted also in previous CASMI contests [2]. Many candidate structures had identical scores with MAGMa+, resulting in the correct matches being given lower ranks according to the evaluation rules. Whereas on average 1098 structures were retained from the structure database based on the parent mass match, only 616 different score values were observed (on average). Team Verdegem will investigate more discriminative scoring options for MAGMa+ in the future.

## Conclusions

This was the first CASMI contest to use a large set of challenges, targeted especially at the automated methods. This decision was taken on the basis of feedback from several representatives at the 2015 Dagstuhl seminar in Computational Metabolomics [51], to allow a statistically more robust comparison of the methods. The decision to provide candidates this year was also on the basis of Dagstuhl discussions, to eliminate the data source as an influence on the contest outcomes and thus focus more on the role of the *in silico *fragmentation approaches themselves.

From the perspective of the organizers, it was a great success to have participants contribute from each of the major different approaches; MetFrag was added internally for the sake of completion as this was not otherwise represented and allows this paper to complement the work in [25] on a different dataset. Very interesting and constructive discussions have resulted from choosing to prepare this article with “all on board” and the post-contest analysis has been instrumental in teasing apart some of the differences between the actual contest results.

The contest winners, **Team Brouard** with CSI:IOKR_A in Category 2 and **Team Kind** with MS-FINDER+MD in Category 3 prove that the latest developments in this field have indeed resulted in great progress in automated structure annotation. Despite the very large candidate sets, the majority of methods achieved around 50% in the Top 10, which is very positive for real-life annotation, especially with an outlook to higher-throughput untargeted analysis. The combination of the Category 2 submissions resulted in even better overall performance than each individual method, indicating the complementarity of the approaches and supporting the potential use of *consensus* fragmentation results as has been shown earlier for fragmenters [12, 52] and also recently for toxicity modeling using a more sophisticated weighting than that attempted here [53]. The role of the metadata and comparison with Category 1 shows that sample context cannot be ignored during identification.

In this contest, few participants used the CASMI training set provided, which was also a suggestion from Dagstuhl. In the end this was too “big” for pure parameter optimization (where a few spectra may suffice), but too small for serious method training. Team Brouard added it to their other training data in their original submissions, while it was used to determine the score weights in the MetFrag entries. Team Vaniya did not use this for MS-FINDER to avoid over-training; Team Allen due to a lack of time. One conclusion from the post-contest evaluation is that future CASMIs could consider providing an extensive, open training dataset (e.g. the GNPS/MassBank collection used by CSI:FID) and ensure all CASMI challenges are absent from this set. This would, however, force all machine-learning approaches to retrain their methods prior to submission. Another option is that the organizers would have to ensure that all challenges are outside all available datasets—which is possible but also difficult with the number of private and closed collections available. A compromise could be to ensure that a sufficient majority of the candidates are outside the “major” mass spectral resources, with some overlap to ensure sufficient challenges are available (finding data sources for CASMI is a challenging task!) and require participants to submit InChIKey lists of their training sets with their submissions; as done with Teams Allen, Brouard and Dührkop post-contest here.

Challenges for future contests remain true unknowns, i.e. substances that are not present in compound databases. This would currently be feasible for manual approaches and was attempted already once in CASMI 2014, Challenges 43–48 [54], albeit with limited success. Automated approaches would need either a metabolite database such as MINEs [50] or structure generation [55], but finding sufficient appropriate data for an automated category will also be a challenge for the contest organizers, let alone the participants! The ability to distinguish stereoisomers using MS/MS alone also remains a challenge for the future that is not yet ripe enough for a CASMI contest; distinguishing (positional) isomers is likely sufficient challenge for the next few years.

The huge improvements in machine learning approaches will continue as more training data becomes available—the more *high quality* data with likewise *high quality* annotations that becomes available in the open data domain will ensure that the best computational people can work on the best identification methods. The complementarity of the chemistry behind MS-FINDER and the machine learning behind CSI shows that developments in both directions will carry the field forward.

The “take home” messages of CASMI 2016 are:

- The latest developments in the field, CSI:IOKR and MS-FINDER were well-deserved winners of Categories 2 and 3, respectively.
- The complementarity of different approaches is clear; combining several *in silico *fragmentation approaches will improve annotation results further.
- The best methods are able to achieve over 30% Top 1 ranks and most methods have the correct candidate in the Top 10 for around 50% of cases using fragmentation information alone, such that the outlook for higher-throughput untargeted annotation for “known unknowns” is very positive.
- This success rate rises to 70% Top 1 ranks (MS-FINDER) and 87% Top 10 ranks (CFM) when including metadata.
- The machine learning approaches clearly improve with larger training data sets—the more high quality annotated, open data that is available, the better they will get.
- Developments that focus on the chemistry such as MS-FINDER are also essential, especially to cover the cases where no training data is available.
- Despite the above, several challenges remain where the simple combinatorial approach of MetFrag and MAGMa still performs best.
- Improved incorporation of experimental “metadata” will increase annotation successes further, especially for large candidate sets.
- Challenges for future contests remain true unknowns, assessing the ability of methods to distinguish positional isomers and eventually also stereoisomers.

Finally, a big thank you to all those who participated in CASMI 2016 in any way, shape or form and keep an eye on the CASMI website [1] for future editions.

## Availability and requirements

- Project name: CASMI
- Project home page: http://www.casmi-contest.org/
- Operating system(s): Platform independent
- Programming language: Various
- License: N/A
- Any restrictions to use by non-academics: none.

## Additional files

**Additional file 1.** This file contains additional content (methods, results and selected spectra) to complement the manuscript. See PDF for details.

**Additional file 2.** *Table A1* ESD file used in MS-FINDER version 1.62 for a total of 220,212 compounds. Additional columns for InChIKey, short InChIKey, exact mass, formula, SMILES are not shown here. The use of N/A and a database identifier represents the presence or absence of a compound in a given database. For example, 1,3-butadiyne is only present in ChEBI database (CHEBI:37820). This ESD file was replaced by a dummy file where all HMDB identifiers were modified to dummy identifiers AV001... AV00n and all other identifiers replaced by -1 or N/A. *Table A2*: Formatted ESD file for CASMI 2016 Category 2 Challenge-001. The first 10 compounds from the candidates list for Challenge-001 are listed above. Columns for InChIKey, short InChIKey, PubChem CID, exact mass, formula, SMILES are shown in this table. Databases from BMDB through PubChem are replaced by dummy information.

## Abbreviations

CASMI: Critical Assessment of Small Molecule Identification
CSI:IOKR: Compound Structure Identification:Input Output Kernel Regression
MS/MS: tandem mass spectrum
ESI: electrospray ionization
HCD: higher-energy collisional dissociation
LC–MS: liquid chromatography coupled to mass spectrometry
$[\hbox {M}{+}\hbox {H}]^{+}$, $[\hbox {M}{-}\hbox {H}]^{-}$: protonated and deprotonated molecular ions
SPLASH: SPectraL hASH
MGF: Mascot Generic Format
SMILES: Simplified Molecular Input Line Entry System
InChI, InChIKey: IUPAC International Chemical Identifier and (hash) key
CSV: comma-separated values
MS1: full scan mass spectrum
RRP: relative ranking position
CFM-ID: Competitive Fragmentation Modeling for Metabolite Identification
NIST: National Institute of Science and Technology (USA)
HMDB: human metabolome database
ChEBI: Chemical Entity of Biological Interest
CSI:FID: Compound Structure Identification:FingerID
IOKR: Input Output Kernel Regression
(Uni-)MKL: (Uniform) Multiple Kernel Learning
CDK: Chemistry Development Kit
GNPS: Global Natural Products Social Networking
SVM: support vector machine
Q-TOF: Quadrupole Time of Flight
HR: hydrogen rearrangement
GUI: graphical user interface
MoNA: MassBank of North America
ESD: Existing Structure Database
CSIDs: ChemSpider Identifiers
RT: retention time
MINEs: Metabolic *In Silico* Network Expansion Databases
EI-MS: electron ionization mass spectrometry
