# Supplementary material for: Critical Assessment of Small Molecule Identification 2016: automated methods
Source: J Cheminform. 2017 Mar 27;9:22. doi: 10.1186/s13321-017-0207-1 (PMC5368104; doi:10.1186/s13321-017-0207-1)
Supplement: Supplementary file 1 — Additional file 1. This file contains additional content (methods, results and selected spectra) to complement the manuscript. See PDF for details. [file 13321_2017_207_MOESM1_ESM.pdf]

Critical Assessment of Small Molecule Identification 2016: Automated Methods

Emma L. Schymanski[1*], Christoph Ruttkies[2], Martin Krauss[3], Céline Brouard[4,5], Tobias Kind[6], Kai Dührkop[7], Felicity Allen[8], Arpana Vaniya[6,9], Dries Verdegem[10], Sebastian Böcker[7], Juho Rousu[4,5], Huibin Shen[4,5], Hiroshi Tsugawa[11], Tanvir Sajed[8], Oliver Fiehn[6,12], Bart Ghesquière[10] and Steffen Neumann[2]

[*]Correspondence:
emma.schymanski@eawag.ch
[1]Eawag: Swiss Federal Institute for Aquatic Science and Technology, Überlandstrasse 133, 8600 Dübendorf, Switzerland
Full list of author information is available at the end of the article

Supporting Information

This document contains additional information for the "Critical Assessment of Small Molecule Identification 2016: Automated Methods" as follows:

- Additional Methods: MS–FINDER and MetFrag
- Additional Results: Retention Time, Comparison with Category 1, Cluster Plots without Training Data
- Selected Mass Spectra – Challenging Challenges
- Additional Plots: Number of Candidates versus Rank, Visualizing Participant Raw Scores

Additional Methods

MS–FINDER

Team Kind (Tobias Kind, Hiroshi Tsugawa, Masanori Arita and Oliver Fiehn) submitted entries to Category 3 using the freely available MS–FINDER version 1.60 [1, 2], MS/MS searching and structure database lookup for confirmation (entry `MS-FINDER+MD`). The full methods (summarized in the main text) are as follows:

First, molecular formulas and structures were determined with MS-FINDER, which was originally developed for the theoretical assignment of fragment substructures to mass spectra. Generally MS-FINDER (http://prime.psc.riken.jp/Metabolomics_Software/) determines molecular formulas using Lewis and Senior checks as well as isotopic abundance information from the precursors ions. This is required to restrict the search space. In this case only MS/MS spectra were provided, meaning molecular formulas could only be calculated with less accuracy. However MS-FINDER utilizes an internal formula database, which prioritizes existing formulas from large chemical databases over less common formulas. The elements C, H, N, O, P, S, F, Cl, Br, I were included (Si was excluded) and 5 ppm mass accuracy for MS1 and 20 ppm for MS2 were assumed. The top 5 molecular formulas were regarded for structure queries. Each formula was then queried in the CASMI candidate lists as well as an internal MS-FINDER structure database. Whereas the CASMI candidate list contained up to 8000 compounds per challenge, the internal MS-FINDER database was compiled from thirteen major biological and environmentally relevant databases (see section *Team Vaniya* below for more details) and was used to prioritize structure lookups. A tree-depth of 2 and relative abundance cutoff of 1% as well as up to 100 possible structures were reported

with MS-FINDER. The score was calculated by the *in silico* fragmenter, which simulated the alpha-cleavage of linear chains with the consideration of hydrogen rearrangement (HR) rules up to three chemical bonds. Multiple bonds (double-, triple-, or cycles) were modeled as penalized single bonds in which hydrogen rearrangements were also utilized. The final score was calculated by the integration of mass accuracy, isotopic ratio, product ion assignment, neutral loss assignment, bond dissociation energy, penalty of fragment linkage, penalty of HR rules, and existence of the compound in the internal MS-FINDER structure databases.

Secondly, MS/MS search was used for further confirmation via the NIST MS Search GUI (`http://chemdata.nist.gov/`) together with major MS/MS databases such as NIST [3], MONA (`http://mona.fiehnlab.ucdavis.edu/`), ReSpect [4] and MassBank [5]. The precursor was set to 5 ppm and product ion search tolerance to 200 ppm. Around 100 out of the 208 candidates had no MS/MS information. For these searches that gave no MS/MS results, a simple similarity search without precursor information was also used, or the precursor window was extended to 100 ppm.

Thirdly, those results that gave overall low hit scores were also cross-referenced with the STOFF-IDENT database of environmentally-relevant substances [6, 7] to obtain information on potential hit candidates. This step was taken because the training set consisted of mostly environmentally relevant compounds.

Team Vaniya (Arpana Vaniya, Stephanie N. Samra, Sajjan S. Mehta, Diego Pedrosa, Hiroshi Tsugawa and Oliver Fiehn) participated in Category 2 using MS-FINDER [1, 2] version 1.62 (entry `MS-FINDER`).

MS/MS spectra were uploaded to MS-FINDER in .msp file format. Precursor m/z, ion mode, mass accuracy of instrument, and precursor type were used as metadata in each file header to populate the fields in MS-FINDER. Further parameter settings were: tree depth of 2, relative abundance cut off of 1 and maximum report number of 100. The default formula finder settings were used: Lewis/Senior rules check, 5 % isotope ratio tolerance, element probability check, element ratio check at common range (99.7 %), elements selected O, N, S, P, F, Cl, Br, I, up to 100 reported results. The only difference to the default values was the mass tolerance, which was set to $\pm$5 ppm mass accuracy as given by the CASMI organizers.

MS-FINDER typically retrieves candidates from an Existing Structure Database (ESD) file located in the Resources sub-folder in the main MS-FINDER folder. The original ESD file contained entries such as title (name), InChIKey, short InChIKey (the first block), PubChem CID, exact mass, formula and SMILES as well as additional database identifiers compiled from 13 databases: HMDB [8, 9], YMDB [10], PubChem [11], SMPDB [12], UNPD [13], ChEBI [14], PlantCyc [15], BMDB [16], KNApSAck [17], FooDB [18], ECMDB [19], DrugBank [20] and T3DB [21].

However, as candidates were provided with CASMI, all structure databases used in MS-FINDER were disabled prior to analysis. For each of the 208 challenges the ESD file was replaced with a formatted ESD file containing information from the candidate lists provided by the CASMI organizers. This ensured that all previous structural information was removed. The ESD files were sorted by mass (least to greatest) to satisfy the binary search criteria used in MS-FINDER. The headers in

the ESD files were kept identical, all PubChem CID entries were changed to -1, all HMDB entries were changed to a dummy database identifier for each candidate compound starting with AV001 to AV00n, (where n was the maximum number of candidates for that challenge), while all other database identifiers were changed to N/A. Example original and formatted ESD files are shown in Additional File 2, Tables A1 and A2 respectively. All local databases were disabled under the parameter settings expect for HMDB; the PubChem online setting was set to "never use it". A batch search of the challenge MS/MS against the challenge candidate list (in the ESD) was performed on the top 500 candidates, to avoid long computational run times. Up to 500 top candidates structures were exported as a text file from MS-FINDER. Scores for automatically matching experimental to virtual spectra were ranked based on mass error, bond dissociation energy, penalties for linkage discrepancies or violating hydrogen rearrangement rules. Final scores and multiple candidate SMILES were reported for 199 challenges for submission to CASMI 2016. Nine challenges could not be processed due to time constraints.

MetFrag

Team Ruttkies (Christoph Ruttkies, Emma Schymanski and Steffen Neumann) submitted internal entries, such that MetFrag2.3 [22] could be evaluated alongside the other methods outside the actual competition, since the organizers could not participate in the contest itself. These entries were also used to investigate the influence of metadata on the competition results. MetFrag command line version 2.3 (available from `http://msbi.ipb-halle.de/~cruttkie/metfrag/MetFrag2.3-CL.jar`) was used to process the challenges, using the MS/MS peak lists and the ChemSpider IDs (CSIDs) of the candidates provided. Several entries were submitted, using different settings as outlined below.

In Category 2, the `MetFrag` submission consisted of the MetFrag fragmentation approach only. The parameters were set to mzppm = 5, mzabs = 0.001 and tree depth = 2. The adduct type was set to $[M{+}H]^+$ (positive) and $[M{-}H]^-$ (negative mode). Unbound candidates (e.g. salts) and those containing non-standard isotopes were filtered out and not considered in the final scoring.

The entry `MetFrag+CFM` took the results lists from `MetFrag` and used these as input for CFM-ID [23] version 2 to retrieve an additional score that was used to calculate the final score as described in [22]. The score weights (w) were optimized on the available training data and chosen using 100 randomly drawn combinations; the combination yielding the highest number of correct Top 1 ranks in the training set (where the answer was known) was selected, such that $w_{\text{MetFrag}} = 0.5793923$ and $w_{\text{CFM-ID}} = 0.4206077$. The weighted sum of the scores was used to create the scores in the final candidate list.

For Category 3, additional metadata was added to the Category 2 entries. Retention time (`RT`) and reference information (`Refs`) was added to the `MetFrag` results to form the `MetFrag+RT+Refs` entry. For the linear retention time model, retention times from the negative and positive training set were used together with the $\log P$ values calculated with the CDK [24]. The correlation is shown in Figure 1. The ChemSpiderReferenceCount was retrieved from the ChemSpider database using the CSIDs given [25]. The best weight combination was chosen out of 1000 randomly

drawn weights (consistent with the above) to yield: Positive: $w_{\mathrm{MetFrag}} = 0.4260182$, $w_{\mathrm{RT}} = 0.2206725$ and $w_{\mathrm{Refs}} = 0.3533094$ and negative: $w_{\mathrm{MetFrag}} = 0.3982628$, $w_{\mathrm{RT}} = 0.2321251$ and $w_{\mathrm{Refs}} = 0.3696120$.

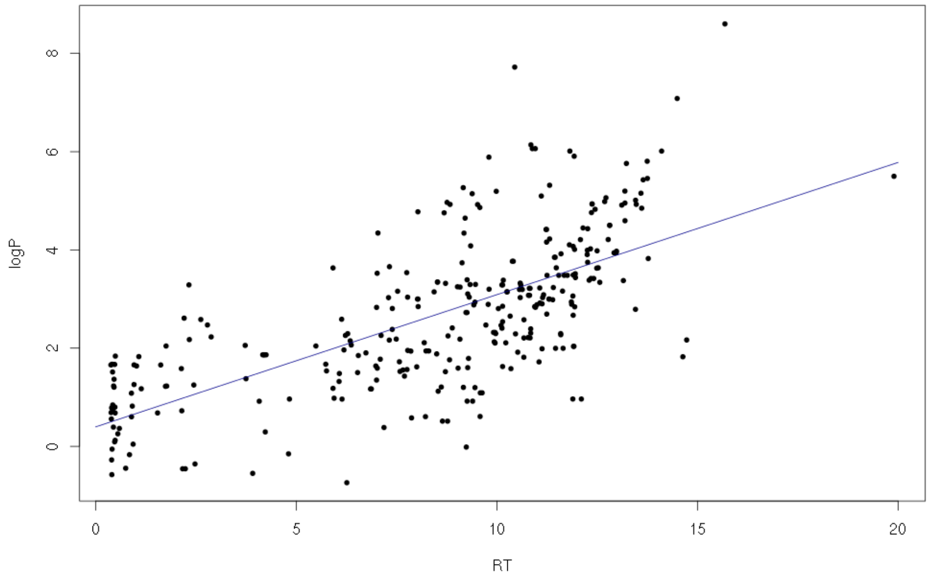

Figure 1 Retention Time Model used in CASMI. Data was taken from the CASMI training set; the correlation coefficient is 0.684. See text for details.

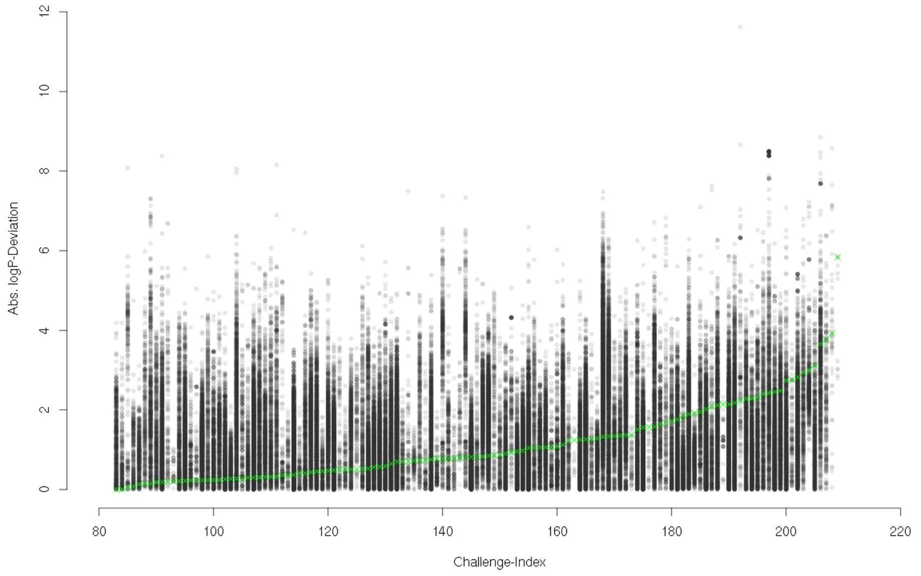

Figure 2 The absolute deviation of the logP (y) for each candidate per challenge (x) from the value predicted from the retention time (positive challenges only). The candidate logPs are shown in greyscale (intensity of the greyscale indicates how many entries with this value), the log P of the correct candidate is shown by the green cross.

The submission `MetFrag+CFM+RT+Refs` used the fragmenter scores from the `MetFrag+CFM` entry and added the reference and retention time information as above. The best weight combination on the training data, out of 1000 randomly drawn weights, was used, yielding: (positive): $w_{\text{MetFrag}} = 0.43807140$, $w_{\text{RT}} = 0.09885304$, $w_{\text{Refs}} = 0.33431292$ and $w_{\text{CFM-ID}} = 0.12876264$. For negative: $w_{\text{MetFrag}} = 0.38728278$, $w_{\text{RT}} = 0.19584541$, $w_{\text{Refs}} = 0.32712506$ and $w_{\text{CFM-ID}} = 0.08974675$.

The submission `MetFrag+CFM+RT+Refs+MoNA` used all the scores mentioned above, with the addition of a structure–spectrum similarity score based on the MetFusion approach [26]. The LC-MS/MS library was downloaded January 2016 from the MassBank of North America (MoNA, `http://mona.fiehnlab.ucdavis.edu/spectra/querytree`). The best of 1000 randomly drawn weights was used (chosen consistent with above) as follows: Positive: $w_{\text{MetFrag}} = 0.16212070$, $w_{\text{RT}} = 0.08104633$, $w_{\text{Refs}} = 0.25308415$, $w_{\text{CFM-ID}} = 0.06701364$, $w_{\text{MetFusionMoNA}} = 0.43673519$ and negative: $w_{\text{MetFrag}} = 0.13587813$, $w_{\text{RT}} = 0.09295245$, $w_{\text{Refs}} = 0.09457464$, $w_{\text{CFM-ID}} = 0.17781439$, $w_{\text{MetFusionMoNA}} = 0.49878039$.

Additional Results

Predicted Retention Time/logP Results

The distribution of predicted retention times, with the placement of the correct candidate for the positive challenges, is shown in Figure 2.

Comparison with Results from Category 1

Challenges 10 – 19 in Category 1 were also present among the Category 2 and 3 challenges, as given in Table 1 in the main text. The results for these challenges, separated by category, are visualized in Figure 3 (positive mode challenges, 10–14) and Figure 4 (negative mode challenges, 15–19). The median rank, number of entries and the range are given in Table 6 in the main article.

Additional Clustering Results - Challenges Absent from All Training Sets

Heat maps from the clustering of all participants and challenges where the correct answer was not present in any CASMI challenge (44 challenges in positive mode, 43 in negative mode) are given in Figures 5 (negative mode) and 6 (positive mode).

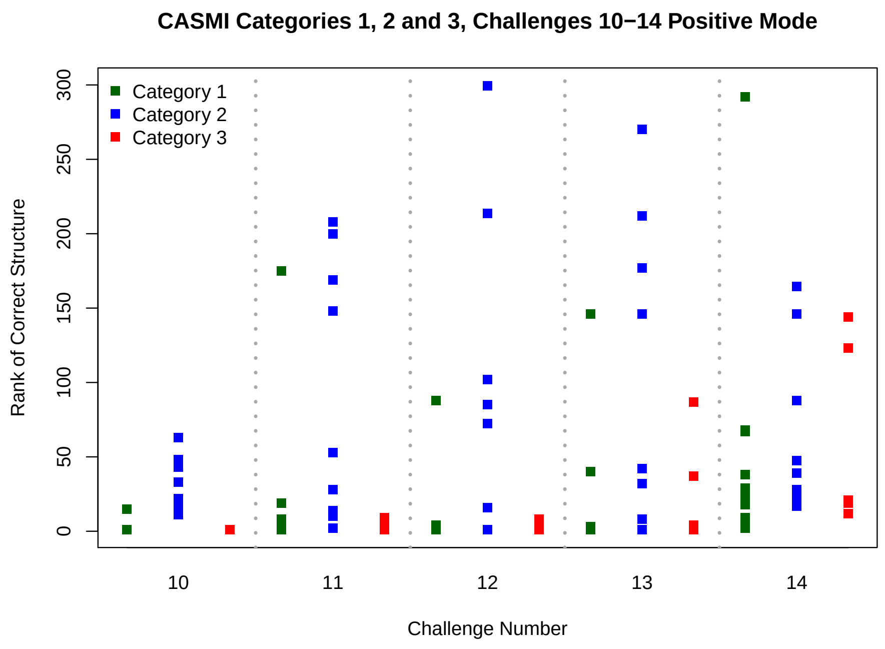

Figure 3 Comparison of Category 1, 2 and 3 entries, Challenges 10 to 14 (Positive mode).

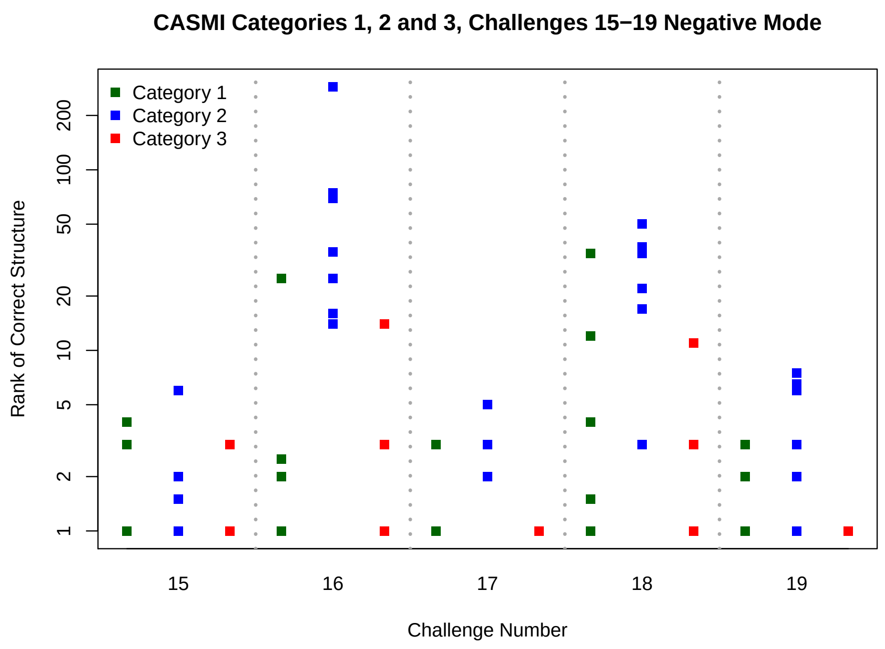

Figure 4 Comparison of Category 1, 2 and 3 entries, Challenges 15 to 19 (Negative mode). Note the logarithmic y scale.

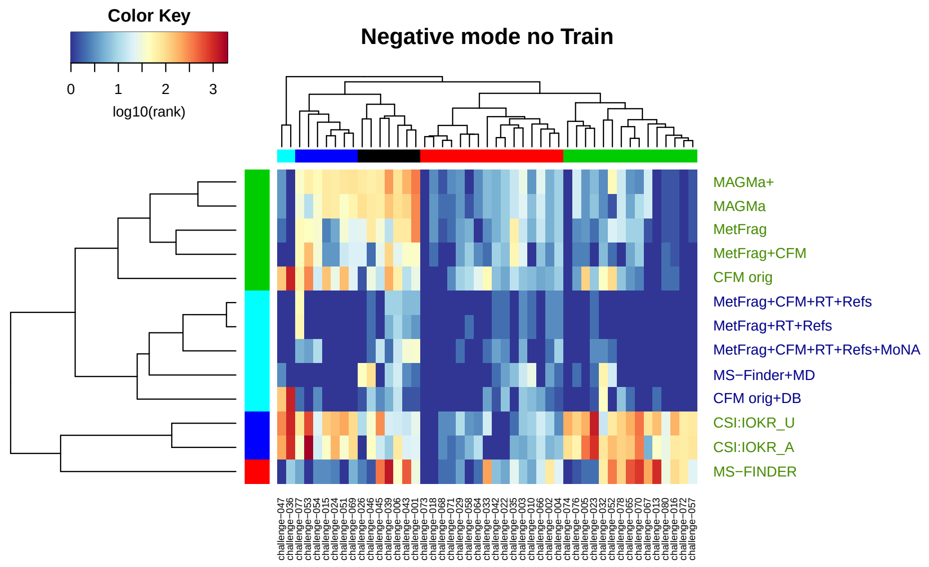

Figure 5 Heat Map of Negative Mode CASMI Challenges not in any training set. Both Category 2 (green labels on the right) and 3 (blue labels) participants are included. Missing values (correct solution missed, or no submission for a challenge) were replaced with the number of candidates for that challenge. Ranks are log-scaled from good (blue) to poor (red). Team Dührkop was omitted as they did not submit for any challenge, while `CSI IOKR_AR` and `CFM_retrain` were omitted as these were identical with their original submissions. An interactive version of this plot is available at http://www.casmi-contest.org/2016/heatmapNegCat2noTrain.html.

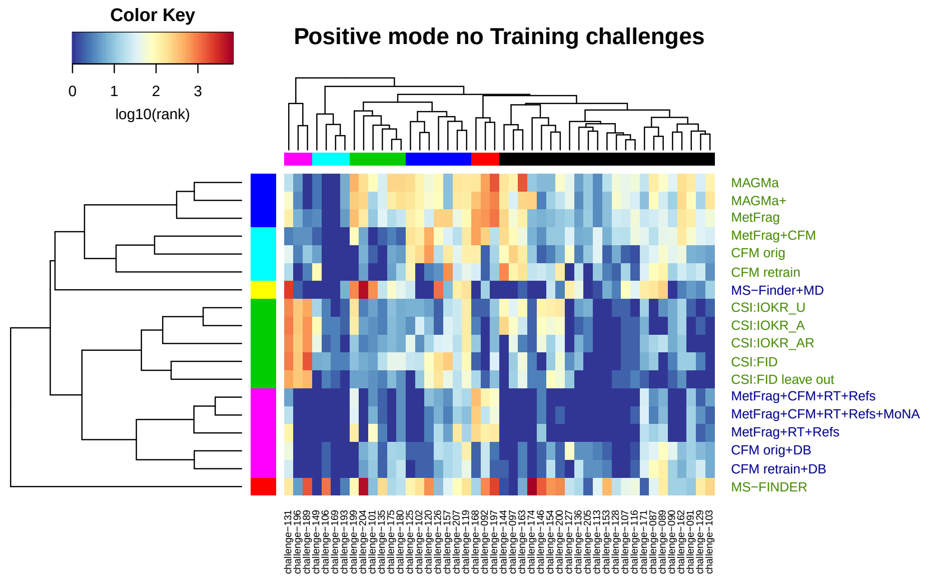

Figure 6 Heat Map of Positive Mode CASMI Challenges not in any training set. Both Category 2 (green labels on the right) and 3 (blue labels) participants are included. Missing values (correct solution missed, or no submission for a challenge) were replaced with the number of candidates for that challenge. Ranks are log-scaled from good (blue) to poor (red). An interactive version of this plot is available at http://www.casmi-contest.org/2016/heatmapPosCat2noTrain.html.

Selected Mass Spectra – Challenging Challenges

Selected spectra for the challenging challenges are as follows:

- Figure 7: Challenge 207 (Challenge 14, Category 1): Abietic acid.
- Figure 8: Challenge 36: Propoxycarbazone.
- Figure 9: Challenge 41: Monobenzyl phthalate.
- Figure 10: Challenge 152: Ethofumesate.
- Figure 11: Challenge 202: Pendimethalin.
- Figure 12: Challenge 178: Michler's ketone.
- Figure 13: Challenge 131: 5-Methyl-1-(propan-2-yl)-1H-indole-2,3dione.
- Figure 14: Challenge 126: 2-(4-Morpholinyl)benzothiazole.
- Figure 15: Challenge 119: 6-Bromo-2(1H)-quinolinone.
- Figure 16: Challenge 184: Medroxyprogesterone.
- Figure 17: Challenge 168: Chlorpropham.
- Figure 18: Challenge 199: Lauric isopropanolamide.
- Figure 19: Challenge 92: 2'-Methylacetanilide.
- Figure 20: Challenge 197: 10-Azabenzo[a]pyrene.

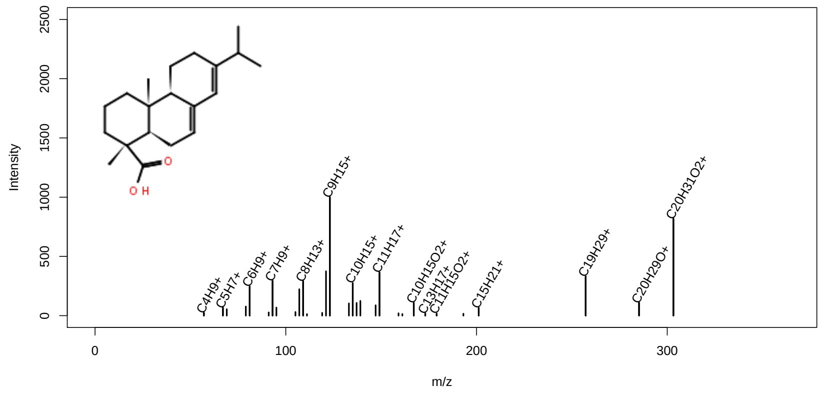

Figure 7 Challenge 14 (Category 1) = Challenge 207 (Category 2 and 3): Abietic acid. Despite the informative mass spectrum and reasonable candidate numbers (581), no method ranked the correct answer in first place. At least one additional substance (isopimeric acid) is very similar, which may explain the results in part.

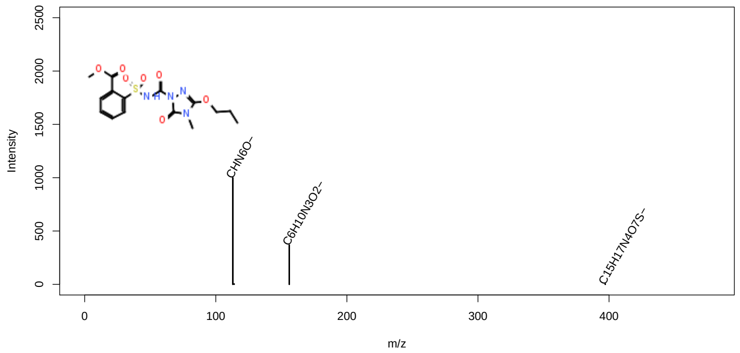

Figure 8 Challenge 36 (Categories 2 and 3): Propoxycarbazone. The relatively uninformative spectrum for a complicated structure explains why the machine learning approaches may have struggled to have good results. This may also be out of the domain of the training sets, as it is a substance more often measured in positive mode. The fragments make sense, but the spectrum has a relatively low information content for such a large molecule.

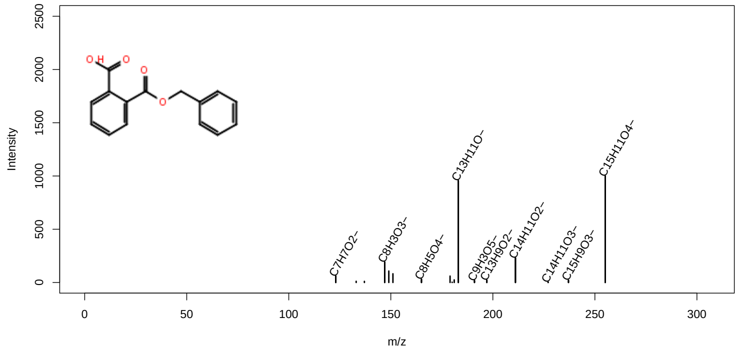

Figure 9 Challenge 41 (Categories 2 and 3): Monobenzyl phthalate. Phthalate spectra are typically dominated by rearrangement reactions; the combinatorial fragmenters struggled with this spectrum but machine learning and metadata approaches performed well, indicating that these substances are well represented in the public domain.

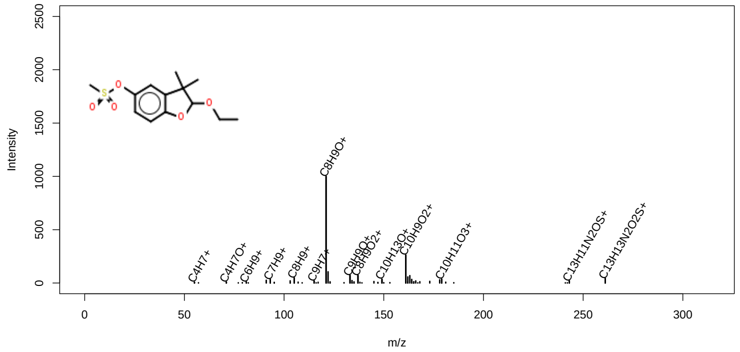

Figure 10 Challenge 152 (Categories 2 and 3): Ethofumesate. Most Category 2 approaches struggled with this challenge, maybe because of the one dominant peak.

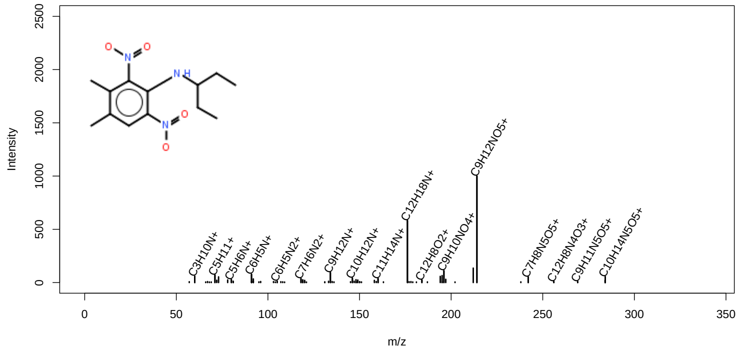

Figure 11 Challenge 202 (Categories 2 and 3): Pendimethalin. Most Category 2 approaches struggled with this challenge, maybe because of only two dominant peaks and the nitro groups, which are prone to rearrangements.

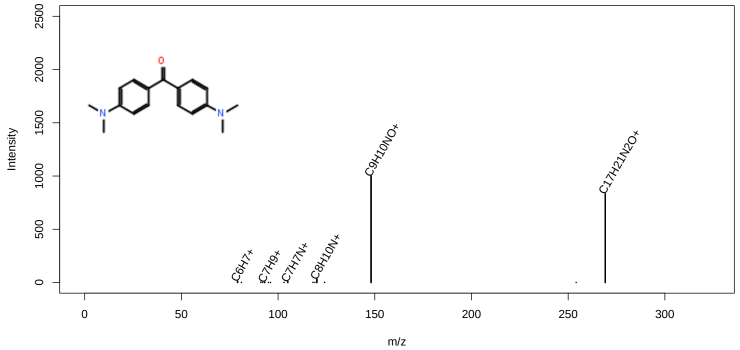

Figure 12 Challenge 178 (Categories 2 and 3): Michler's ketone. Most Category 2 approaches struggled with this challenge, likely because of the one dominant non-precursor peak (as the molecule is symmetric).

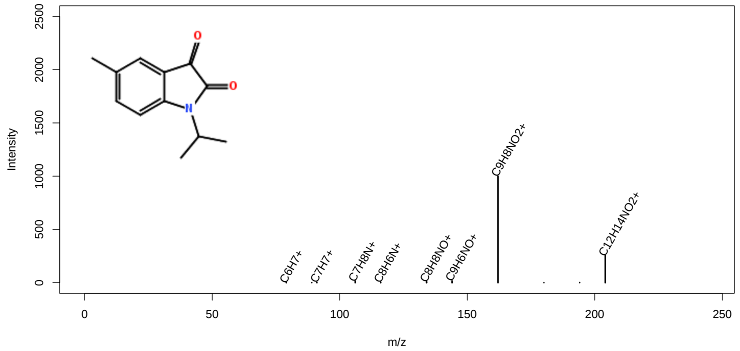

Figure 13 Challenge 131 (Categories 2 and 3): 5-Methyl-1-(propan-2-yl)-1H-indole-2,3dione. Most approaches struggled with this challenge, likely because of the one dominant peak corresponding with the isopropyl group loss.

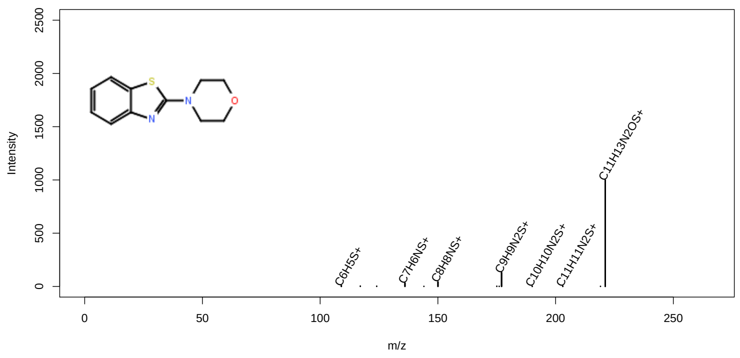

Figure 14 Challenge 126 (Categories 2 and 3): 2-(4-Morpholinyl)benzothiazole. Most approaches struggled with this challenge, maybe because of small peaks and aromatic nitrogens.

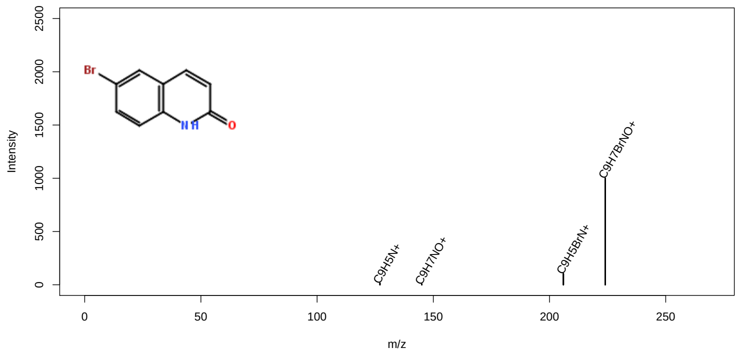

Figure 15 Challenge 119 (Categories 2 and 3): 6-Bromo-2(1H)-quinolinone. Most approaches struggled with this challenge, which is not surprising considering the uninformative spectrum, with few, low intensity peaks where the main peak is a Br loss.

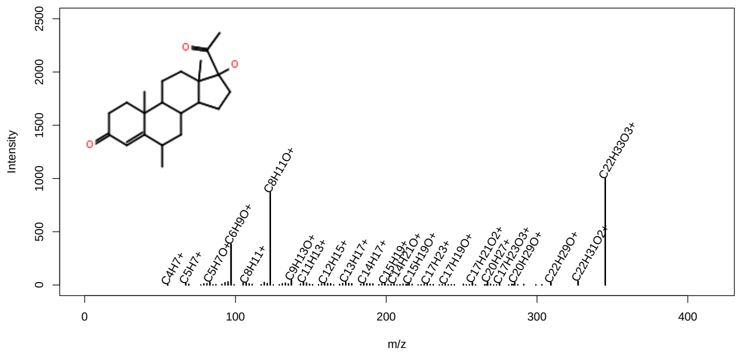

Figure 16 Challenge 184 (Categories 2 and 3): Medroxyprogesterone. The machine learners did well, metadata failed. Many peaks are present, but two dominate. This is a typical steroid spectrum and there are likely to be many similar structures.

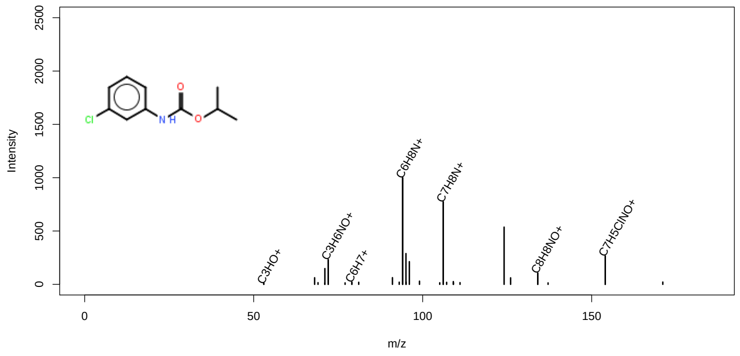

Figure 17 Challenge 168 (Categories 2 and 3): Chlorpropham. The machine learners did well, but metadata failed despite a nice spectrum and it is ranked 2nd in ChemSpider by references. Involves breaking of an amide bond.

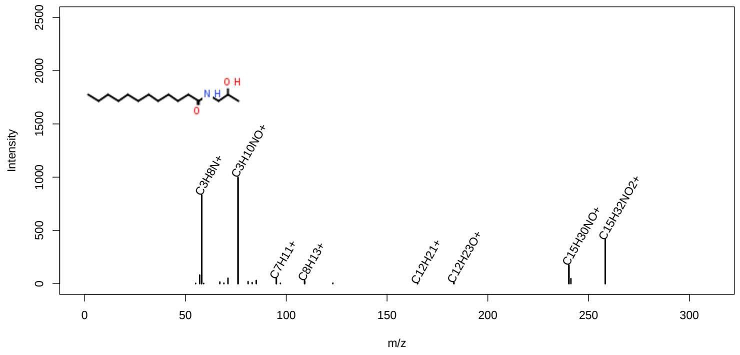

Figure 18 Challenge 199 (Categories 2 and 3): Lauric isopropanolamide. The machine learners did well, metadata failed. There are many structures with similar numbers of references in ChemSpider. This also involves a breaking of an amide bond.

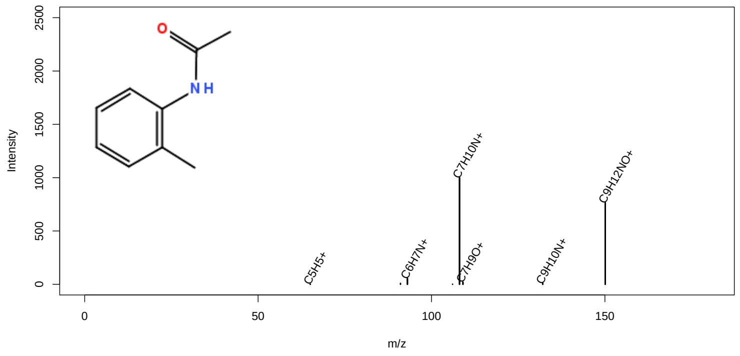

Figure 19 Challenge 92 (Categories 2 and 3): 2'-Methylacetanilide. The machine learners did well, metadata failed. Spectrum is dominated by one main peak. Again this involves an amide bond, but many structures have these present.

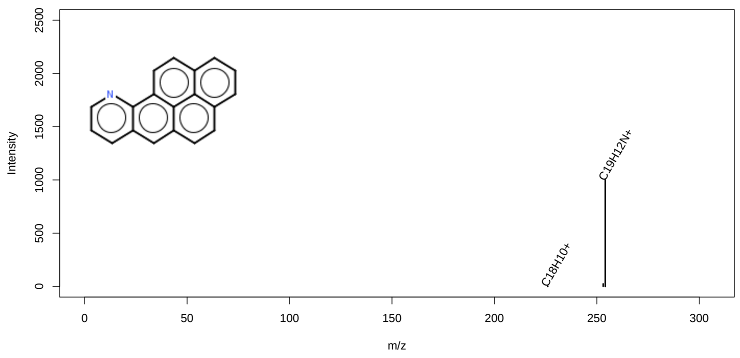

Figure 20 Challenge 197 (Categories 2 and 3): 10-Azabenzo[a]pyrene. The machine learners did well, metadata failed. The spectrum is very uninformative, diagnostic for this substance class - this backs up the ability of the machine learners to learn properties.

Additional Plots

Number of Candidates versus Rank

Figure 21 shows the number of candidates for each challenge, ordered by the number of candidates versus the results for all CASMI entries (during and post–contest). The red line shows the number of candidates, the light blue line the median rank, the dark blue line the smoothed mean rank (over all entries) and dark blue the smoothed median rank. The “X”s show the number of submissions where the candidate was ranked first (Top 1) per challenge, which is a maximum of 18 (shown by the dotted line at y=18). The thicker black line shows the smoothed Top 1 results. The coloured dots show the rank of the correct candidate for each challenge (legend to the left). All in all this plot shows that there is little relationship between the candidate numbers and the rank, in fact the lowest Top 1 counts and highest median and mean ranks were observed for the middle range of candidate numbers (between 200 and 1000 candidates). A trend is observed for low candidate numbers, as would be expected, but surprisingly the smoothed median rank is lower at higher candidate numbers.

Visualizing Participant Raw Scores

The following plots show all candidate scores as submitted by the participants, per challenge for one submission per participant and category to CASMI. These were chosen to best demonstrate the results and reveal great differences in the way the methods treat the raw data. The arrangement was chosen to group given plots together. All scores are shown as small dots, the correct score is shown with a larger green dot. All candidate scores above the correct solution are shown in red, and the highest score as blue dot. If the top score corresponds to the correct solution in green, there are no red dots, and also no blue circle.

The selected plots are, in order (Category 2 only first, followed by the methods that participated in both categories, with Category 2 submission above Category 3): Figure 22: `MAGMa+`, Figure 23: `CSI:IOKR_A`, Figure 24: `CSI:FID`, Figure 25: `CFM_orig`, Figure 26: `CFM_retrain+DB`, Figure 27: `MS-FINDER`, Figure 28: `MS-FINDER+MD`, Figure 29: `MetFrag`, Figure 30: `MetFrag+CFM+RT+Refs+MoNA`.

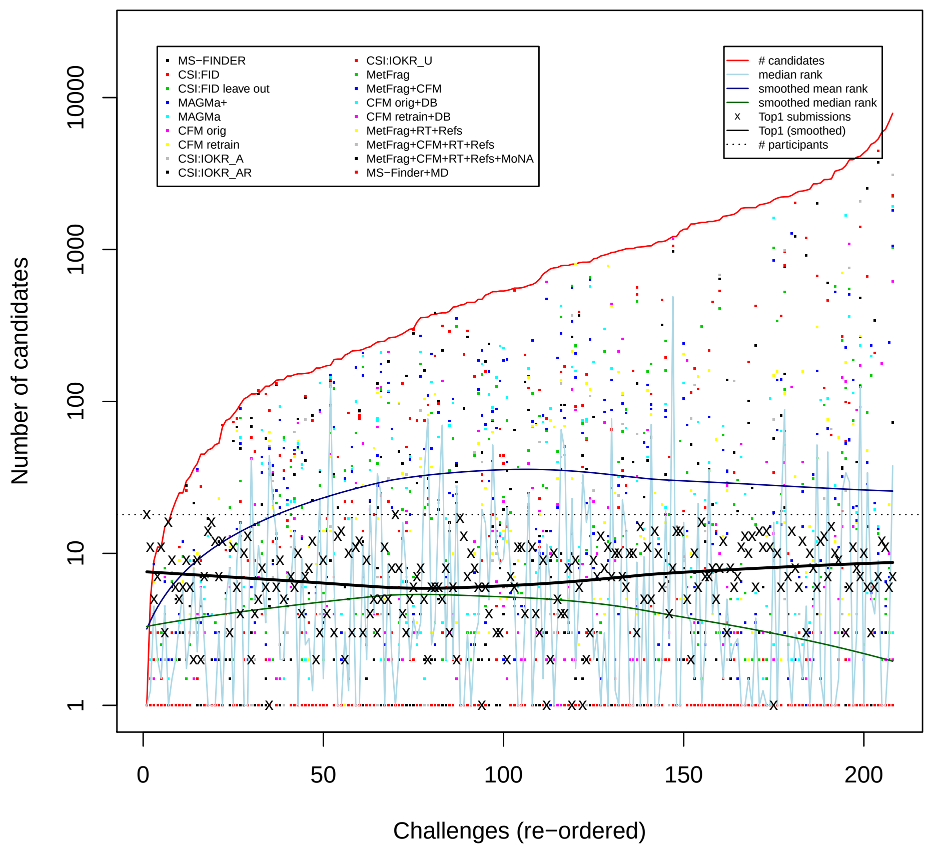

Figure 21 The number of candidates per challenge (ordered by number of candidates) versus the results for all CASMI 2016 entries. See text for details.

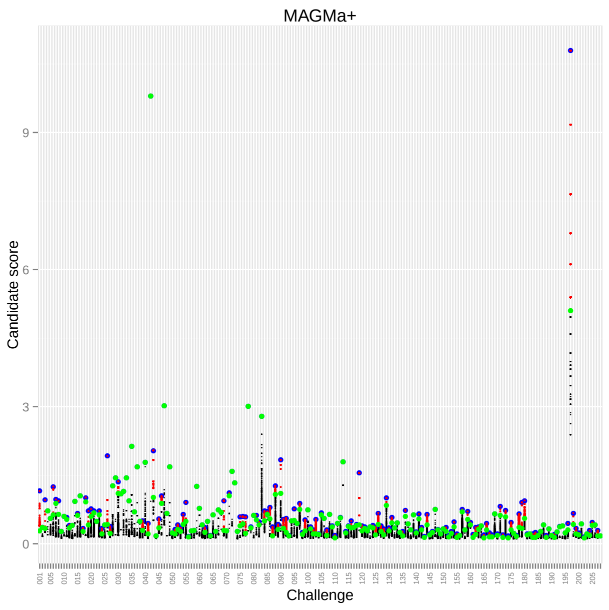

Figure 22 Raw scores per challenge for MAGMa+. Green dot: score of correct candidate, blue dot: highest score (if not green), candidates with scores above correct candidate are red, others black.

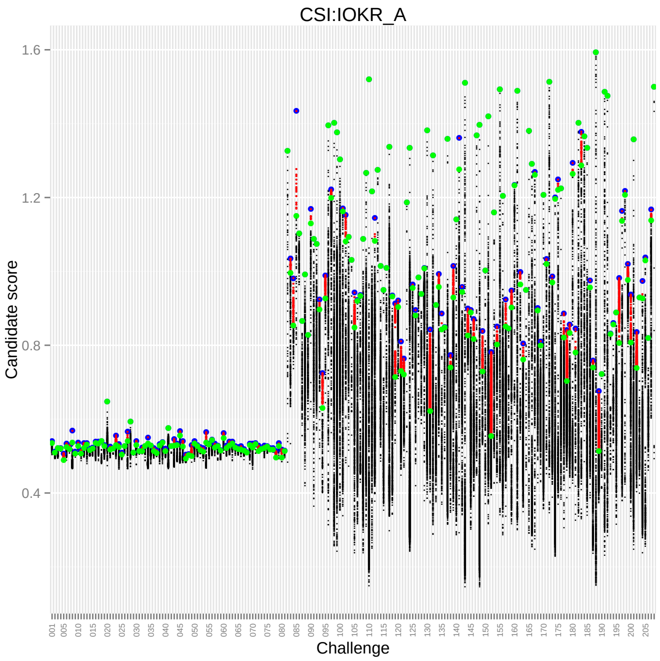

Figure 23 Raw scores per challenge for `CSI:IOKR_A`. Green dot: score of correct candidate, blue dot: highest score (if not green), candidates with scores above correct candidate are red, others black. Note: 0.5 was added to all raw scores to have positive values. The parameter σ controls the raw score variance and was tuned seperately for positive and negative mode. The discrepancy in the scores may be due to the different amount of training data available for each mode.

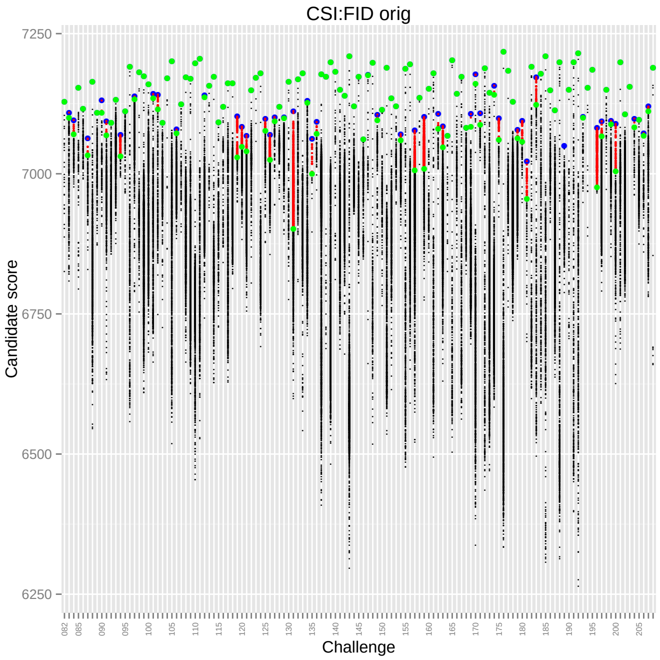

Figure 24 Raw scores per challenge for `CSI:FID`. Green dot: score of correct candidate, blue dot: highest score (if not green), candidates with scores above correct candidate are red, others black.

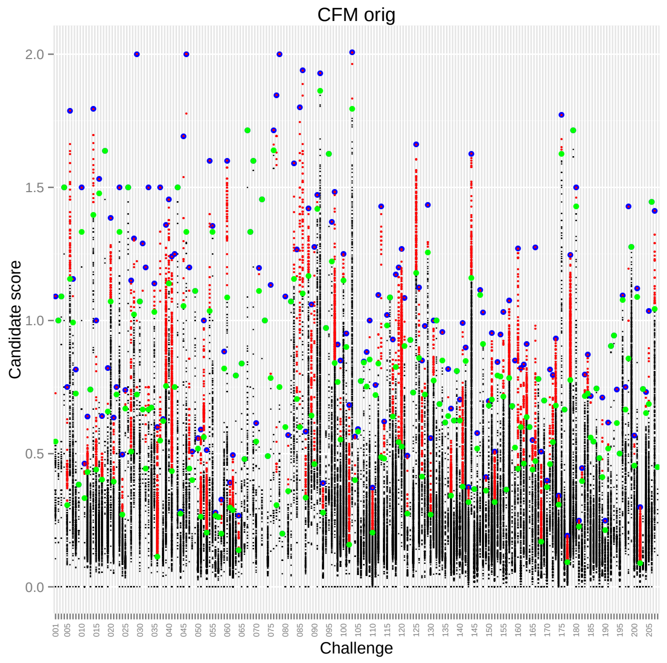

Figure 25 Raw scores per challenge for `CFM_orig`. Green dot: score of correct candidate, blue dot: highest score (if not green), candidates with scores above correct candidate are red, others black.

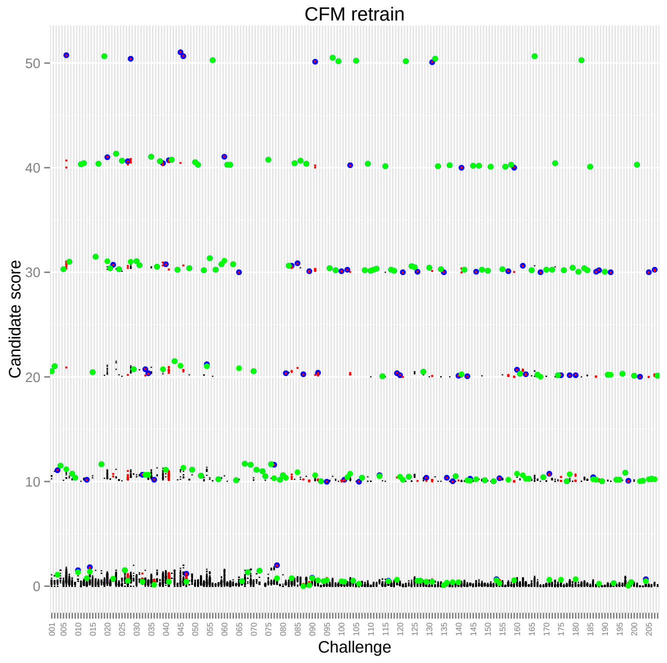

Figure 26 Raw scores per challenge for `CFM_retrain+DB`. Green dot: score of correct candidate, blue dot: highest score (if not green), candidates with scores above correct candidate are red, others black.

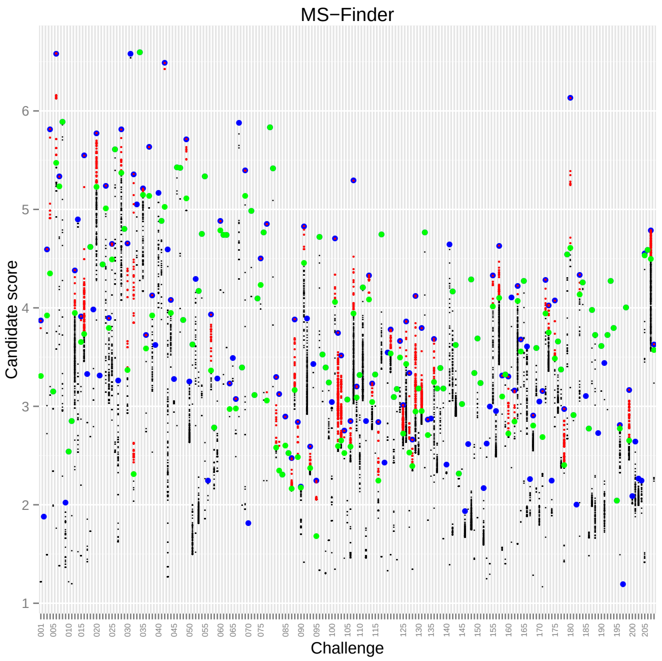

Figure 27 Raw scores per challenge for MS-FINDER. Green dot: score of correct candidate, blue dot: highest score (if not green), candidates with scores above correct candidate are red, others black.

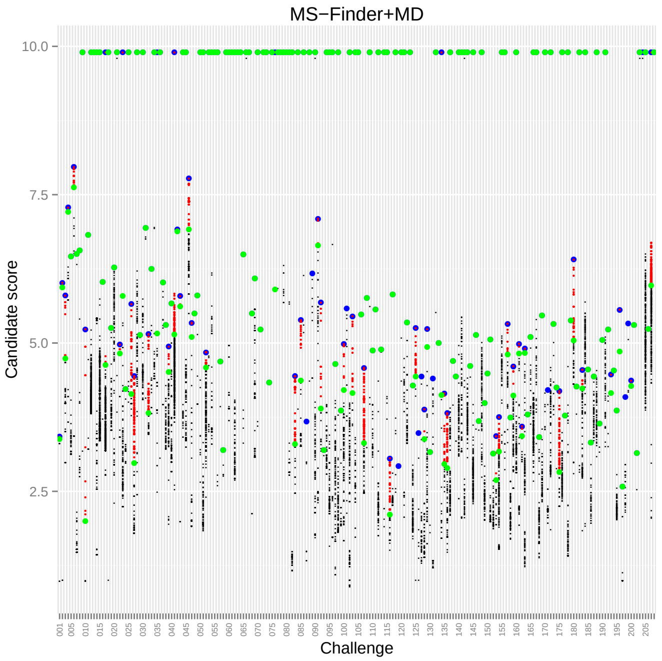

Figure 28 Raw scores per challenge for MS-FINDER+MD. Green dot: score of correct candidate, blue dot: highest score (if not green), candidates with scores above correct candidate are red, others black.

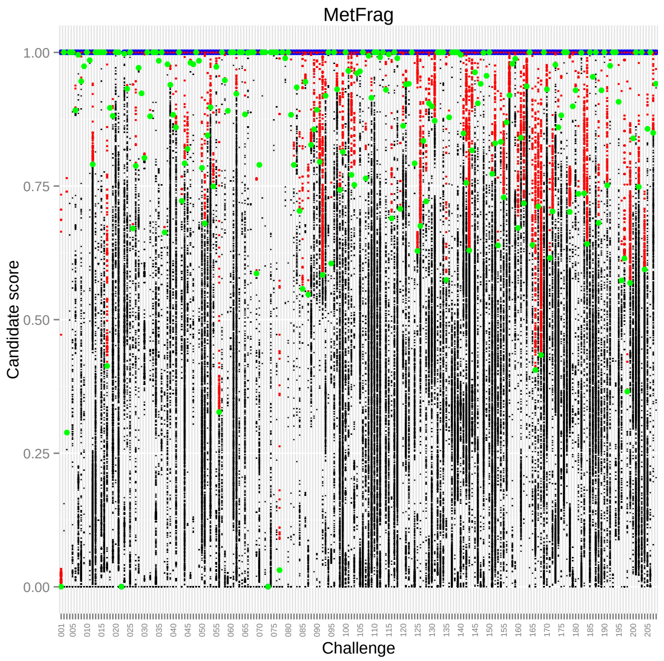

Figure 29 Raw scores per challenge for `MetFrag`. Green dot: score of correct candidate, blue dot: highest score (if not green), candidates with scores above correct candidate are red, others black.

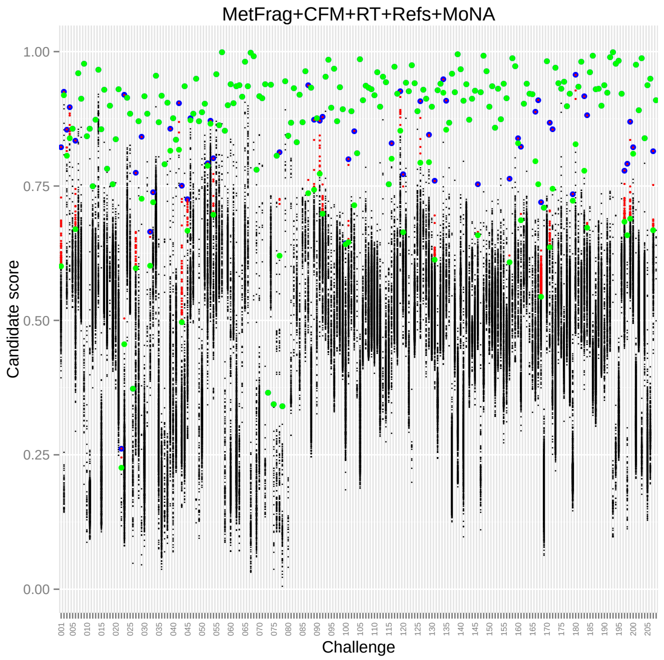

Figure 30 Raw scores per challenge for `MetFrag+CFM+RT+Refs+MoNA`. Green dot: score of correct candidate, blue dot: highest score (if not green), candidates with scores above correct candidate are red, others black.

Author details
[1]Eawag: Swiss Federal Institute for Aquatic Science and Technology, Überlandstrasse 133, 8600 Dübendorf, Switzerland. [2]Leibniz Institute of Plant Biochemistry, Department of Stress and Developmental Biology, Weinberg 3, 06120 Halle, Germany. [3]UFZ: Helmholtz Centre for Environmental Research, Department of Effect–Directed Analysis, Permoserstrasse 15, 04318 Leipzig, Germany. [4]Department of Computer Science, Aalto University, , Espoo, Finland. [5]Helsinki Institute for Information Technology, , Espoo, Finland. [6]University of California Davis, West Coast Metabolomics Center and Genome Center, 451 Health Sciences Drive, 95616 Davis, CA, USA. [7]Chair of Bioinformatics, Friedrich-Schiller-University, Jena, , Jena, Germany. [8]Department of Computing Science, University of Alberta, Canada, , Alberta, Canada. [9]University of California Davis, Department of Chemistry, One Shields Avenue, 95616 Davis, CA, USA. [10]Metabolomics Expertise Center, Vesalius Research Center (VRC), VIB, KU Leuven – University of Leuven, 3000 Louvain, Belgium. [11] RIKEN Center for Sustainable Resource Science (CSRS), 1-7-22 Suehiro-cho, 230-0045 Tsurumi-ku, Yokohama, Kanagawa, Japan. [12] Department of Biochemistry, Faculty of Sciences, King Abdulaziz University, Jeddah, Saudi Arabia.

References

1. Tsugawa, H., Kind, T., Nakabayashi, R., Yukihira, D., Tanaka, W., Cajka, T., Saito, K., Fiehn, O., Arita, M.: Hydrogen rearrangement rules: Computational MS/MS fragmentation and structure elucidation using MS–FINDER software. Analytical Chemistry **88**(16), 7946–7958 (2016)
2. Tsugawa, H., et al.: MS–FINDER. Accessed 8 December 2016. http://prime.psc.riken.jp/Metabolomics_Software/MS-FINDER/index.html
3. NIST/EPA/NIH: NIST Mass Spectral Library 2014 Edition. U.S. Secretary of Commerce, USA
4. Sawada, Y., Nakabayashi, R., Yamada, Y., Suzuki, M., Sato, M., Sakata, A., Akiyama, K., Sakurai, T., Matsuda, F., Aoki, T., *et al.*: RIKEN tandem mass spectral database (ReSpect) for phytochemicals: a plant-specific MS/MS-based data resource and database. Phytochemistry **82**, 38–45 (2012)
5. Horai, H., Arita, M., Kanaya, S., Nihei, Y., Ikeda, T., Suwa, K., Ojima, Y., Tanaka, K., Tanaka, S., Aoshima, K., Oda, Y., Kakazu, Y., Kusano, M., Tohge, T., Matsuda, F., Sawada, Y., Hirai, M.Y., Nakanishi, H., Ikeda, K., Akimoto, N., Maoka, T., Takahashi, H., Ara, T., Sakurai, N., Suzuki, H., Shibata, D., Neumann, S., Iida, T., Tanaka, K., Funatsu, K., Matsuura, F., Soga, T., Taguchi, R., Saito, K., Nishioka, T.: MassBank: A public repository for sharing mass spectral data for life sciences. Journal of Mass Spectrometry **45**, 703–714 (2010)
6. LfU: Bayerisches Landesamt für Umwelt: STOFF-IDENT (login Required). Accessed 13 June 2016. http://bb-x-stoffident.hswt.de/stoffidentjpa/app
7. NORMAN Association: NORMAN Suspect List Exchange. Accessed 8 December 2016. http://www.norman-network.com/?q=node/236
8. Wishart, D.S., Jewison, T., Guo, A.C., Wilson, M., Knox, C., Liu, Y., Djoumbou, Y., Mandal, R., Aziat, F., Dong, E., et al.: HMDB 3.0—the Human Metabolome Database in 2013. Nucleic Acids Research, 1065 (2012)
9. Wishart, D.S., Tzur, D., Knox, C., Eisner, R., Guo, A.C., Young, N., Cheng, D., Jewell, K., Arndt, D., Sawhney, S., *et al.*: Hmdb: the human metabolome database. Nucleic Acids Research **35**(suppl 1), 521–526 (2007)
10. Jewison, T., Knox, C., Neveu, V., Djoumbou, Y., Guo, A.C., Lee, J., Liu, P., Mandal, R., Krishnamurthy, R., Sinelnikov, I., et al.: Ymdb: the yeast metabolome database. Nucleic acids research, 916 (2011)
11. Bolton, E.E., Wang, Y., Thiessen, P.A., Bryant, S.H.: Pubchem: integrated platform of small molecules and biological activities. Annual reports in computational chemistry **4**, 217–241 (2008)
12. Frolkis, A., Knox, C., Lim, E., Jewison, T., Law, V., Hau, D.D., Liu, P., Gautam, B., Ly, S., Guo, A.C., *et al.*: Smpdb: the small molecule pathway database. Nucleic acids research **38**(suppl 1), 480–487 (2010)
13. Gu, J., Gui, Y., Chen, L., Yuan, G., Lu, H.-Z., Xu, X.: Use of natural products as chemical library for drug discovery and network pharmacology. PLoS one **8**(4), 62839 (2013)
14. Degtyarenko, K., De Matos, P., Ennis, M., Hastings, J., Zbinden, M., McNaught, A., Alcántara, R., Darsow, M., Guedj, M., Ashburner, M.: ChEBI: a database and ontology for chemical entities of biological interest. Nucleic acids research **36**(suppl 1), 344–350 (2008)
15. PMN Plant Metabolic Network. Accessed 8 December 2016. http://www.plantcyc.org/
16. BMDB: Bovine Metabolome Database. Accessed 8 December 2016. http://www.cowmetdb.ca/cgi-bin/browse.cgi
17. Afendi, F.M., Okada, T., Yamazaki, M., Hirai-Morita, A., Nakamura, Y., Nakamura, K., Ikeda, S., Takahashi, H., Altaf-Ul-Amin, M., Darusman, L.K., *et al.*: Knapsack family databases: integrated metabolite–plant species databases for multifaceted plant research. Plant and Cell Physiology **53**(2), 1–1 (2012)
18. Wishart, D.S.: FooDB. Accessed 8 December 2016. http://foodb.ca/
19. Guo, A.C., Jewison, T., Wilson, M., Liu, Y., Knox, C., Djoumbou, Y., Lo, P., Mandal, R., Krishnamurthy, R., Wishart, D.S.: Ecmdb: the e. coli metabolome database. Nucleic acids research **41**(D1), 625–630 (2013)
20. Wishart, D.S., Knox, C., Guo, A.C., Cheng, D., Shrivastava, S., Tzur, D., Gautam, B., Hassanali, M.: DrugBank: A knowledgebase for drugs, drug actions and drug targets. Nucleic Acids Research **36**(suppl 1), 901–906 (2008)
21. Wishart, D., Arndt, D., Pon, A., Sajed, T., Guo, A.C., Djoumbou, Y., Knox, C., Wilson, M., Liang, Y., Grant, J., *et al.*: T3db: the toxic exposome database. Nucleic acids research **43**(D1), 928–934 (2015)
22. Ruttkies, C., Schymanski, E.L., Wolf, S., Hollender, J., Neumann, S.: MetFrag relaunched: Incorporating strategies beyond in silico fragmentation. Journal of Cheminformatics **8**(1), 1 (2016)
23. Allen, F., Greiner, R., Wishart, D.: Competitive fragmentation modeling of ESI-MS/MS spectra for putative metabolite identification. Metabolomics (2014). doi:10.1007/s11306-014-0676-4
24. Steinbeck, C., Han, Y., Kuhn, S., Horlacher, O., Luttmann, E., Willighagen, E.: The Chemistry Development Kit (CDK): An open-source java library for chemo- and bioinformatics. Journal of Chemical Information and Computer Sciences **43**(2), 493–500 (2003)
25. Royal Society of Chemistry: ChemSpider. http://www.chemspider.com/
26. Gerlich, M., Neumann, S.: MetFusion: Integration of compound identification strategies. Journal of Mass Spectrometry **48**(3), 291–298 (2013). doi:10.1002/jms.3123
